# Identification and Evaluation of Natural Compounds as Potential Inhibitors of NS2B-NS3 Zika Virus Protease: A Computational Approach

**DOI:** 10.1007/s12033-024-01357-6

**Published:** 2024-12-28

**Authors:** Nada Anede, Mebarka Ouassaf, Kannan R. R. Rengasamy, Shafi Ullah Khan, Bader Y. Alhatlani

**Affiliations:** 1 Group of Computational and Medicinal Chemistry, LMCE Laboratory, University of Biskra, Biskra, Algeria; 2 Laboratory of Natural Products and Medicinal Chemistry (LNPMC), Saveetha Medical College and Hospital, Saveetha Institute of Medical and Technical Sciences (SIMATS), Thandalam, Chennai, 602105 India; 3 Centre of Excellence for Pharmaceutical Sciences, North-West University, Potchefstroom, South Africa; 4 Normandie Univ, Université de Caen Normandie, Inserm U1086 ANTICIPE (Interdisciplinary Research Unit for Cancer Prevention and Treatment), 14076 Caen, France; 5 Comprehensive Cancer Center François Baclesse, UNICANCER, 14076 Caen, France; 6 Unit of Scientific Research, Applied College, Qassim University, Buraydah, 52571 Saudi Arabia

**Keywords:** Zika virus, Molecular dynamics simulation, Antiviral agents, Viral nonstructural proteins

## Abstract

**Abstract:**

The Zika virus (ZIKV), an arbovirus within the Flavivirus genus, is associated with severe neurological complications, including Guillain-Barré syndrome in affected individuals and microcephaly in infants born to infected mothers. With no approved vaccines or antiviral treatments available, there is an urgent need for effective therapeutic options. This study aimed to identify new natural compounds with inhibitory potential against the NS2B-NS3 protease (PDB ID: 5LC0), an essential enzyme in viral replication. An e-pharmacophore model was generated using a five-point (ADDRR) feature approach in the PHASE module of Schrodinger and used for the virtual screening of 26,689 natural compounds from the PubChem database. The screening yielded 14,277 prioritized compounds based on fitness scores, further refined through extra precision (XP) docking in GLIDE, resulting in 24 compounds. Eight top hits were selected following ADME analysis with SwissADME, and toxicity screening with ProTox-II identified four non-toxic lead candidates. Molecular dynamic simulations confirmed the stability of the three most promising leads, CID 44418637, CID 163078083, and CID 68734190, with binding affinities of − 7.721, − 8.226, and − 8.307 kcal/mol, respectively. MM/GBSA analysis revealed that Compounds 68734190 (− 50.192 kcal/mol) and 163078083 (− 49.947 kcal/mol) possess superior binding affinities to the ZIKV NS2B-NS3 protease compared to the reference compound (− 38.347 kcal/mol). Given their natural origin, these compounds may offer safer options to mitigate severe ZIKV-related symptoms while providing a favourable safety and pharmacokinetic profile. This study lays the groundwork for developing targeted ZIKV therapies, potentially addressing a significant unmet need in public health by reducing the incidence of ZIKV-related complications. Further experimental validation is required to confirm efficacy and address potential development challenges.

**Graphical abstract:**

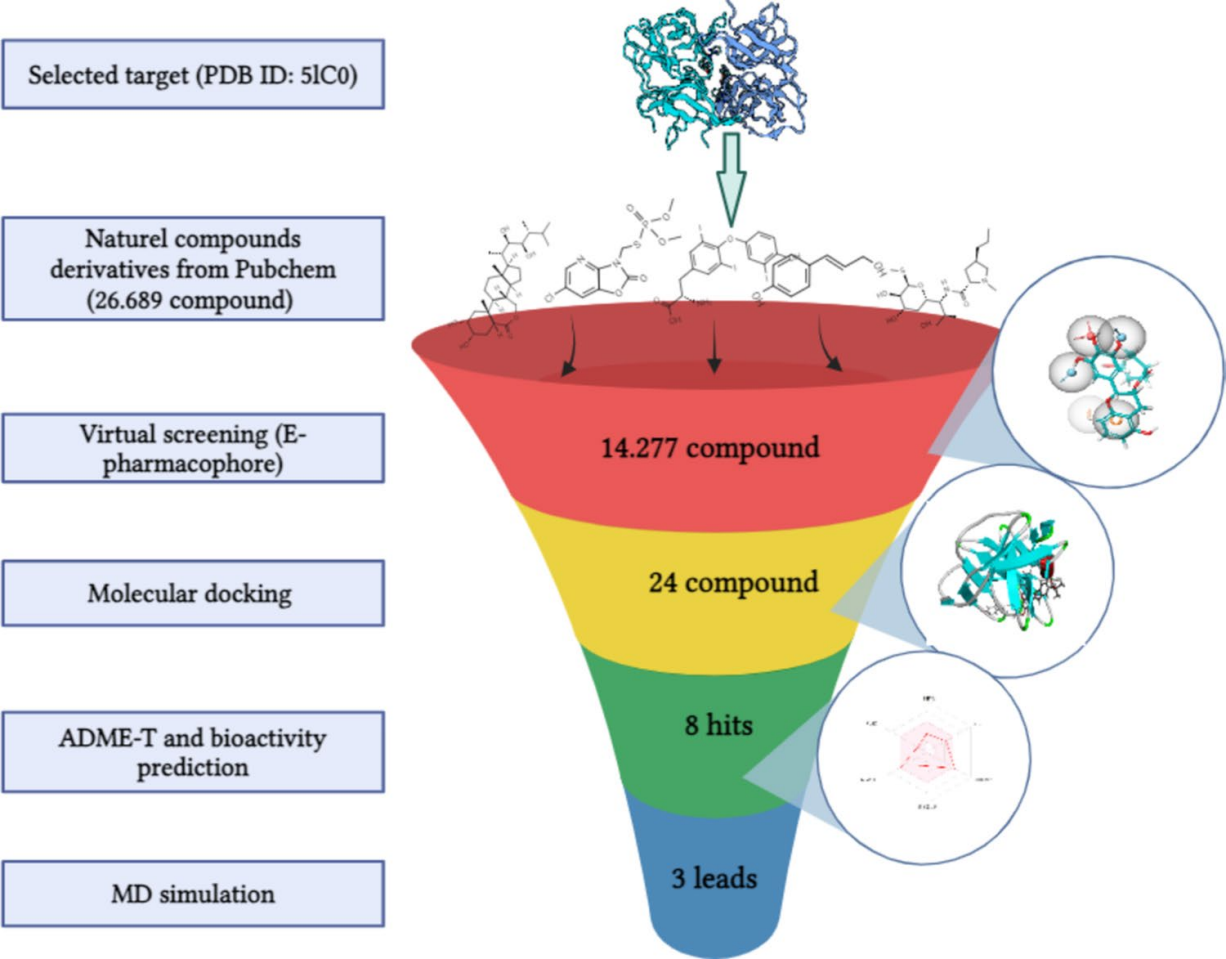

**Supplementary Information:**

The online version contains supplementary material available at 10.1007/s12033-024-01357-6.

## Introduction

Although the Zika virus (ZIKV) has been known for over seventy years, it remains a significant threat, with the potential for a global pandemic exacerbated by climate change, which may alter the dynamics of the mosquitoes that effectively transmit the virus [1, 2]. As of February 2022, the World Health Organization (WHO) reports no authorized vaccine or therapeutic alternative for treating Zika or its infections; management is currently focused on alleviating symptoms [3].

The Zika virus is named after the Zika Forest in Uganda, where it was first isolated in April 1947 from a rhesus monkey. The first recorded human infection occurred in a Nigerian female in 1954 [4, 5]. ZIKV primarily transmits to humans through Aedes mosquitoes, making it a mosquito-borne virus similar to dengue virus (DENV), West Nile virus (WNV), Japanese encephalitis virus (JEV), yellow fever virus (YFV), and tick-borne encephalitis virus (TBEV) [6]. The main vectors for ZIKV transmission are the mosquito species *Aedes aegypti* and *Aedes albopictus* [7].

ZIKV belongs to the *Flaviviridae* family and the *Flavivirus* genus of positive RNA-stranded viruses. Its genome is approximately 10,794 nucleotides long, encoding 3419 amino acids, and consists of an icosahedral, lipid-enveloped virion [8, 9]. The viral genome encodes three structural proteins: capsid (C), the precursor of the membrane protein (prM), and envelope (E), along with seven nonstructural proteins (NS1, NS2A, NS2B, NS3, NS4A, NS4B, and NS5) that are crucial for viral replication [10]. Among these, the NS2B-NS3 protease complex plays a pivotal role in the posttranslational modification of the viral polyprotein, making it a key target for anti-Zika drug development [11].

The NS2B-NS3 protease complex is essential for Zika virus replication, with NS2B functioning as a cofactor that enhances the enzymatic activity of NS3, which is critical for viral polyprotein processing [12–14]. However, developing inhibitors for this protease poses challenges due to the enzyme's structural complexity and similarity to human proteases, complicating the design of selective and effective inhibitors [15, 16]. Given the urgent need for safe Zika treatments and the lengthy process of experimental drug discovery, computational approaches provide a valuable and efficient alternative for screening large libraries of compounds to identify promising therapeutic candidates.

Recent studies have utilized computational methods such as molecular docking, quantitative structure–activity relationship (QSAR) models, and high-throughput screening to discover potential inhibitors of the NS2B-NS3 protease [17–19]. Nevertheless, many of these studies have overlooked natural compounds, which are typically associated with safer profiles and fewer side effects—a critical consideration for treatments that may be used in pregnant women due to Zika's serious risks during pregnancy [20].

**Fig. 1:**
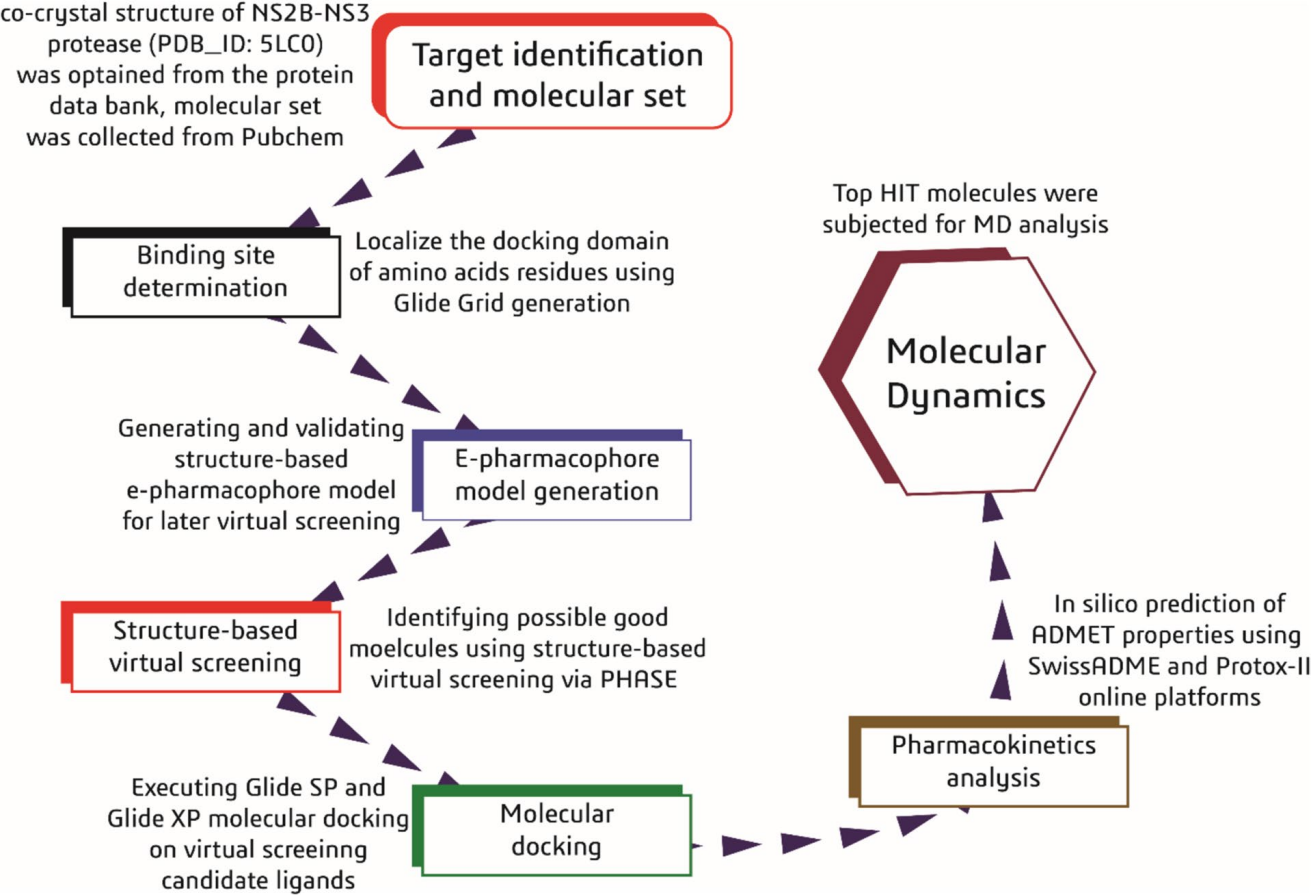
An overview of different phases applied for identifying NS2B-NS3 Zika virus protease inhibitors

Our study emphasized natural compounds and their analogs as potential safe inhibitors for the NS2B-NS3 protease. By employing a comprehensive computational approach that included e-pharmacophore modelling, virtual screening, molecular docking, and molecular dynamics simulations, we identified promising candidates based on their ADME (absorption, distribution, metabolism, and excretion) and toxicity profiles and binding affinities. This focus on natural compounds and computational screening lays a solid foundation for developing selective and effective anti-Zika therapies, as illustrated in Fig. 1.

## Materials and Methods

### Preparation of the Protein

The X-ray crystal structure of Zika virus NS2B-NS3 protease under code 5LC0 was imported from the RCSB Protein Data Bank (PDB) with a 2.7 Å resolution [21]. The protein structure is a dimer complex with a co-crystal boronate inhibitor in each monomer. The boronate inhibit reversibly the ZIKV NS2B-NS3 protease with IC50 = 0.25 ± 0.02 µM and Ki = 0.040 ± 0.006 µM [21]. One of the monomers (chain A) was saved and prepared utilizing the Protein Preparation Wizard of Maestro 12.5, as shown in Fig. A1. The preparation process contained three procedures. The first was pre-processing, which consisted of different options where the hydrogens were added, the bond orders were adjusted, the water molecule was removed, chains were missing sides, and loops were missed. The following procedure is to review and modify. In this step, the multimeric complex was simplified by removing chain B and its inhibitor ligand. Finally, the last procedure, named refine; in this folder, the PH equal 7 was determined, and the force field applied is OPLS3e to minimize energy until a root mean square mean deviation (RMSD) of 0.37 Å was obtained.

### Receptor Grid Generation

The receptor grid-generating panel of Glide was utilized to create the grid box of the prepared protein. This step is necessary and obligatory before docking ligands, and it is impossible to dock a ligand without the grid generation step [22]. The binding pocket site determined with coordinates reported by [23] X = 81.82, Y = 51.89, and Z = 153.92 was produced at the center of the active site. The receptor Vander Waal’s radii atoms scaled by 1.00 Å to reduce the potential for non-polar parts of the receptor with a partial charge of 0.25. The dock ligands length was 20 Å. For identifying the important residues that contribute to substrate binding, the boronate inhibitor was picked in the box as reference ligand, as result the including residues are Asp1075, His1051, Asp83, Gly1151, Asn1152, Ser81, Val1154, Asp1129, Tyr1130, Tyr1150, Tyr1161, Ala1132, Val1036, Val1052, Gly1133, Tyr1161, Pro1131, Thr1134, Gly1037, Ser1135, Gly1153, Phe84, Ser85, Val1072, Gly82, Trp1050 this residues was showed by Discovery Studio 2021 Visualizer (Fig. A2).

**Fig. 2:**
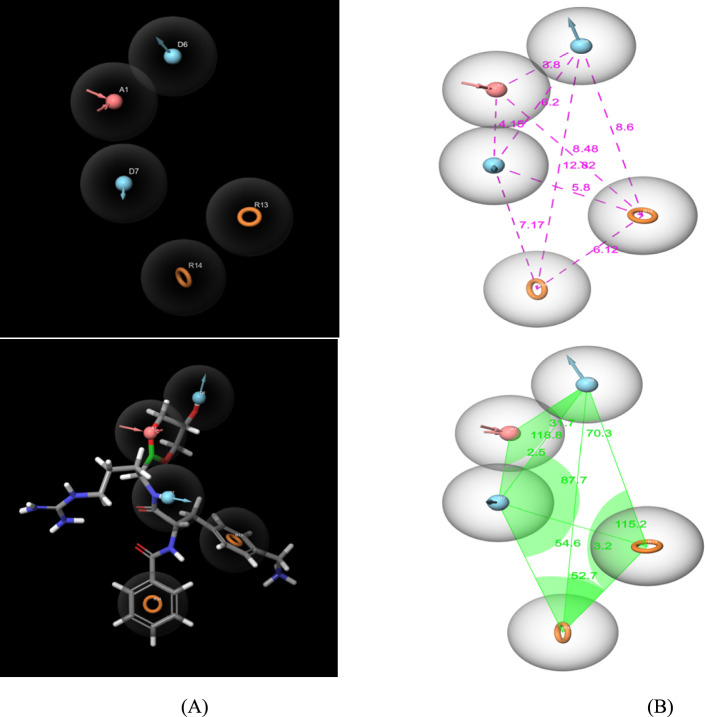
**a** The generated e-pharmacophore (ADDRR) of ZIKV NS2B-NS3 protease with and without boronate inhibitor. **b** Inter-features, angles and distances between five pharmacophoric points

### Ligand Preparation

The 26,689 compounds have been chosen from derivatives of different families of natural sources. Most are flavonoids (Epigallocatechin gallate, Rutin, Myricetin, Hydroxypanduratin, Schaftoside, Silychristin). Other compounds are alkaloids (Berberine) and dipeptides (Carnosine). These compounds were selected from various studies [23–29]. The structure file of all molecules was obtained from the PubChem database in sdf format. The approved compounds filtered by PubChem followed two rules, Lipinski and Veber, to highlight the chosen compounds with perfect properties and equilibrium. Then, the downloaded compounds were prepared using the Ligprep collection tools in Maestro. The Ligprep module produces high-quality ligands with correct chirality, low energy, and three-dimensional structures, as well as ring confirmation for all input ligands. The tautomers and ionization states were defined at pH 7.0 ± 2.0. The OPLS3e force field was utilized to minimize the energies of each ligand.

### Molecular Docking

Molecular docking was used because it is one of the most utilized methods in structure-based drug design. This method predicts the interactions between ligands and the protein's active site [30, 31]. The docking analysis of prepared ligands (14,277 compounds matched the pharmacophore model) was performed into the predicted active site of the NS2B-NS3 protease using the GLIDE suite from Schrödinger. This looks for good interactions between ligands and proteins and aids in improving the affinity of the binding by determining the presence of certain compounds. SP and XP docking were used to dock the ligands with the active site, and the scaling factor was set to 0.80 with a partial charge of 0.15. In the first, Standard-Precision (SP) docking was used for large sets of ligands. After that, only the top-scoring ligands were selected and docked in Extra-Precision (XP) mode for more accuracy with the highest score. The result of the NS2B-NS3 protease and boronate inhibitor complex was then employed to generate an e-pharmacophore model.

### Docking Validation

Maestro's enrichment calculator panel was used to validate the docking method. At first, the actives and decoys file was prepared with Ligprep. After that, the file was docked into the studied protease NS2B-NS3, and the SP docking was utilized. The active compounds were obtained from previous studies that showed a moderate IC50 against NS2B-NS3 Zikv protease [24, 32]. For decoys, one of the active compounds was used to generate decoys from a PubChem database with a similarity of 20%. The decoys and actives files were in sdf format.

### Generation of E-Pharmacophore Model

Energy-based pharmacophore (E-pharmacophore) generates energetically optimized, structure-based pharmacophores that can be used to screen a large dataset of ligands rapidly [33, 34]. The PHASE module of Schrödinger version 4.3 was used to develop an e-pharmacophore model of the NS2B-NS3 protease with a boronate inhibitor. Phase contains six different Standard chemical features such as hydrophobic group (H), acceptor and donor of hydrogen bond (A/D), ring aromatic (R), and negatively and positively ionizable (N/P) [35]. Before creating the e-pharmacophore, the Glide XP module was used at first to dock the bound ligand centroid in the binding site of the protein of this research to pick out the best-ranked position for the e-pharmacophore model generation. This made it easier to measure and rank the sites according to their energy levels. Finally, using the pharmacophore model allowed us to reduce the number of compounds from 26,689 to 14,277. For docking, ligands with appropriate chemical properties that matched at least five features on the generated e-pharmacophore model were selected to identify potential NS2B-NS3 inhibitors.

### ADME Analysis

ADME has pharmacokinetic properties that refer to a pharmaceutical compound's absorption, distribution, metabolism, and excretion. These properties have an essential role in the development of a drug candidate. In addition to the compounds with the right acceptable pharmacokinetics characteristics at a therapeutic dose and sufficiently active against the target, it is classified as a high-quality drug candidate[33, 36]. The best-screened hits ADME profile was determined using the SWISSADME platform to filter the top compounds to find new inhibitors. In this study, the best compounds were evaluated using different standard databases to examine pharmaceutical relevant such as Physicochemical Properties (molecular weight, number of rotatable bonds, number of acceptors and donor H-bond and TPSA), Lipophilicity (logP), Water Solubility (logS), Pharmacokinetics ( GI absorption, BBB permeant, P-gp substrate, CYP1A2 inhibitor, CYP2C19 inhibitor, CYP2C9 inhibitor, CYP2D6 inhibitor, CYP3A4 inhibitor), Druglikeness ( Lipinski, Veber, Bioavailability Score).

### Toxicity and Bioactivity Prediction

The toxicity of a chemical substance is the degree to which it can be poisonous or damage an organism or a part of the organism. Prediction of toxicity levels is an important step in drug discovery and chemical safety that aids in picking out compounds with the highest potential for safe and efficient usage in humans, and it can reduce the time and preclinical and clinical testing costs [37]. The toxicity was evaluated using ProTox-ll, a virtual online lab that allows you to predict the toxicity of small molecules by inserting the SMILE format of the ligand. Six toxicity values were predicted from the ProTox-ll platform: Hepatotoxicity, Carcinogenicity, Immunotoxicity, Mutagenicity, Cytotoxicity and LD50 of the screened compounds. An antiviral prediction was added using the PASSonline web tool to support our study on the selected safe molecules. This website is used to evaluate the biological activity of virtual compounds, and it can determine their bioactivity with a confidence level of approximately 90%. This step is the last stage in screening the compounds to choose the top leads selected for Molecular dynamic simulations.

### Molecular Dynamic

Molecular dynamics simulations were carried out using the Desmond software from Schrödinger LLC, based in New York, NY, USA. We used the NPT ensemble, setting the temperature at 300 K and pressure at 1 bar. These conditions were regulated using the Martyna-Tuckerman-Klein chain coupling method with a 2.0 ps coupling constant for pressure and the Nosé-Hoover chain coupling method for temperature [38]. The simulations employed the OPLS_2005 forcefield. The particle mesh Ewald (PME) approach was applied to calculate long-distance electrostatic forces, with a Coulomb interaction cutoff radius set at 9.0 Å [39]. Water molecules were modelled using the simple point charge (SPC) method [40]. We computed nonbonded forces, updating the short-term forces at each step and the long-term forces at every third step [41]. The simulation duration was 100 ns, with a 1 ps relaxation time for the top three hits compounds and reference compounds. Data trajectories were recorded for subsequent analysis. We assessed the dynamics and interactions of the ligands with the target protein using Desmond’s Simulation Interaction Diagram (SID) feature. The stability of these simulations was continuously monitored by examining the RMSD, RMSF and the Contacts of Protein–Ligand of the positions of ligand and protein atoms throughout the simulation. Following the molecular dynamics (MD) simulations, the binding free energies of the Reference Compound, Compound 68,734,190, Compound 44,418,637, and Compound 163,078,083 were calculated using the Molecular Mechanics-Generalized Born Surface Area (MM/GBSA) method. This analysis was performed on the ZIKV NS2B-NS3 protease-ligand complexes trajectories using the Prime module within the Maestro software suite. The Prime module employs the MM/GBSA continuum solvent model, which incorporates the OPLS4 force field, VSGB solvent model, and rotamer search algorithms, to determine binding free energies from the MD conformations.

All 1000 frames from each MD simulation trajectory were included in the post-MD MM/GBSA calculations to ensure a thorough analysis of binding interactions despite the associated computational cost. The following equation was used to calculate the binding free energies:

$$\Delta {\text{E}} = {\text{EC}} - {\text{ER}} - {\text{EL}}$$

where ΔE represents the binding free energy, EC is the total energy of the protein–ligand complex, ER is the total energy of the receptor (ZIKV NS2B-NS3 protease), and EL is the total energy of the ligand. The OPLS4 force field and VSGB solvent model were consistently applied throughout the calculations.

## Results

### E-Pharmacophore Model Generation

The e-pharmacophore model was employed due to its qualitative nature, which facilitates the identification of new compounds with specific biological activities and aids in screening inhibitors with a consistent orientation [34, 42]. In this study, the prepared protein–ligand complex (NS2B-NS3 protease with a boronate inhibitor) was a reference for generating the e-pharmacophore hypothesis using the Phase and Glide programs for simulation.

The generated e-pharmacophore hypothesis model encompasses five features, represented as ADDRR: two aromatic rings (R13, R14), two hydrogen bond donors (D6, D7), and one hydrogen bond acceptor (A1). The e-pharmacophore model, both with and without the boronate ligand, is illustrated in Fig. 2a, while Fig. 2b shows the distances between the pharmacophoric points.

Based on a fitness score greater than 1.0 for structure-based virtual screening, the initial set of 26,698 compounds underwent the LigPrep phase, resulting in a refined list of 14,277 compounds. This reduction was achieved by applying the e-pharmacophore model and selecting compounds that matched at least five features of our hypothesis.

### Docking Validation

Validation of the docking process is a crucial step in the screening workflow. In this phase, 25 active compounds known for their inhibitory activity against NS2B-NS3 were used alongside 1,000 inactive compounds obtained from the PubChem database. Various statistical parameters were calculated to evaluate the docking method, including ROC (Receiver Operating Characteristic curve), BEDROC (Boltzmann-enhanced discrimination of ROC), AUC (Area Under the Curve), and RIE (Robust Initial Enhancement), as detailed in the supplementary file in Tables B1–B4 [43].

The ROC value ranges from 0 to 1, with our study yielding a result of 0.91, indicating strong performance in ranking active compounds more effectively than inactive ones [43]. This finding is further supported by the ROC plot presented in Fig. B1. The AUC value of 0.89 indicates that 89% of true positive results were accurately identified using the docking method, demonstrating its efficacy in selecting and scoring active compounds ahead of the decoys [42].

The RIE value was also found to be 8.10, a figure commonly preferred for validation. The BEDROC values measured at various tuning parameters were (α = 8.0/0.667), (α = 20.0/0.512), and (α = 100.9/0.137). The latter represents the likelihood that an active compound is ranked above inactive compounds [44]. These results confirm that the docking method is effective and validated for virtual screening.

### Molecular Docking

Molecular docking analysis was performed using the Glide module of Schrödinger, focusing on the binding site of the NS2B-NS3 ZIKV protease. The 14,277 compounds identified from the Phase screening underwent docking studies utilizing two processes to evaluate top-ranked ligands.

Initially, the boronate inhibitor (CID 137348520) was selected as a reference ligand, achieving a docking XP score of − 5.492 kcal/mol. Subsequently, all compounds were subjected to SP docking, where only those with a docking score of − 5.9 kcal/mol or lower were advanced to XP docking. Ultimately, 24 compounds with XP docking scores ranging from − 6.668 to − 9.166 kcal/mol were selected as the top-ranked molecules, as presented in Table B6.

For interaction analysis, only the highest-scoring compound from each group of natural compound derivatives was included in this study, resulting in eight molecules. The docking scores for these compounds are summarized in Table 1.

**Table 1:** The XP docking score of the best-docked ligands

The group of the compound	Compound CID	Docking score	Glide emodel	XP Gscore
Epigallocatechin gallate	166479806	− 8.454	− 60.980	− 8.459
Berberine	166625687	− 6.992	− 74.687	− 7.110
Rutin	68734254	− 7.750	− 61.548	− 7.752
Carnosine	44418637	− 7.336	− 56.995	− 7.721
Myricetin	163078083	− 8.226	− 48.204	− 8.226
Hydroxypanduratin	42605183	− 8.325	− 58.037	− 8.330
Schaftoside	68734190	− 8.305	− 63.170	− 8.307
Silychristin	58178603	− 9.165	− 68.635	− 9.166
Boronate inhibitor	137348520	− 4.817	− 62.971	− 5.492

The compounds with high docking scores, including CID 58178603, CID 166479806, CID 42605183, CID 68734190, and CID 163078083, demonstrate significant alignment with the pharmacophore features. Notably, CID 58178603 achieved the highest docking score of − 9.166 kcal/mol, indicating excellent compatibility with all pharmacophore features. In contrast, compounds such as CID 166625687, CID 68734254, and CID 44418637 exhibited lower docking scores, suggesting a lack of alignment with some pharmacophore features.

**Fig. 3:**
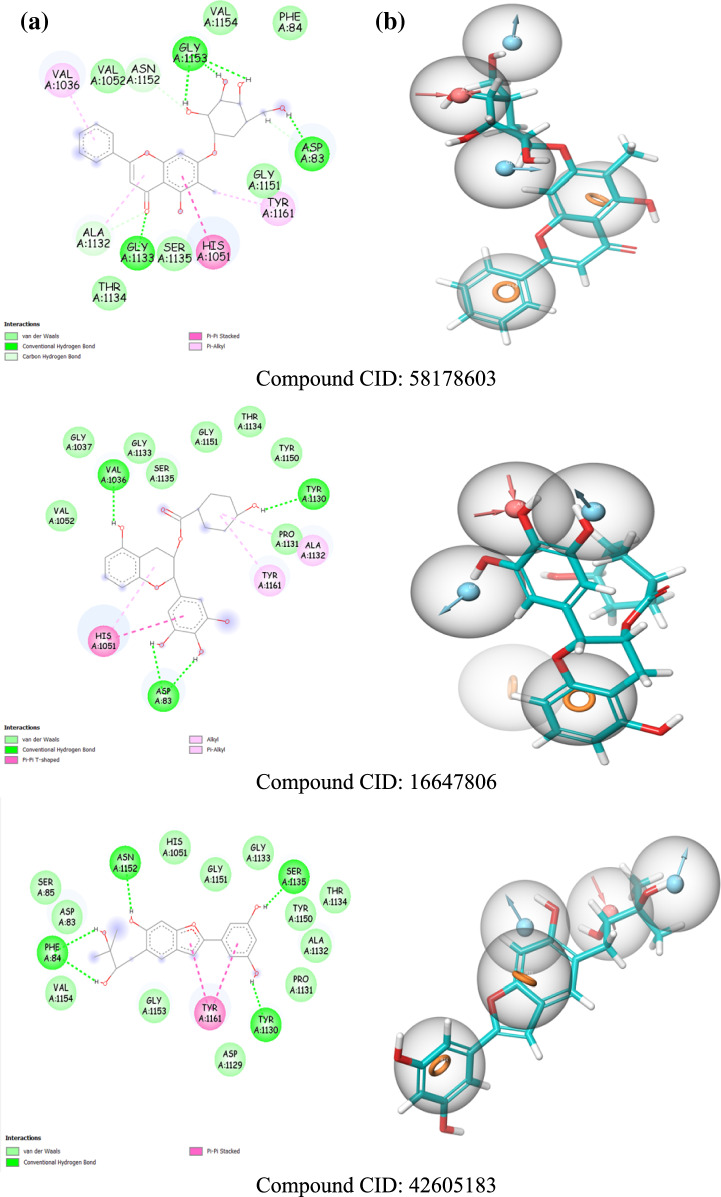

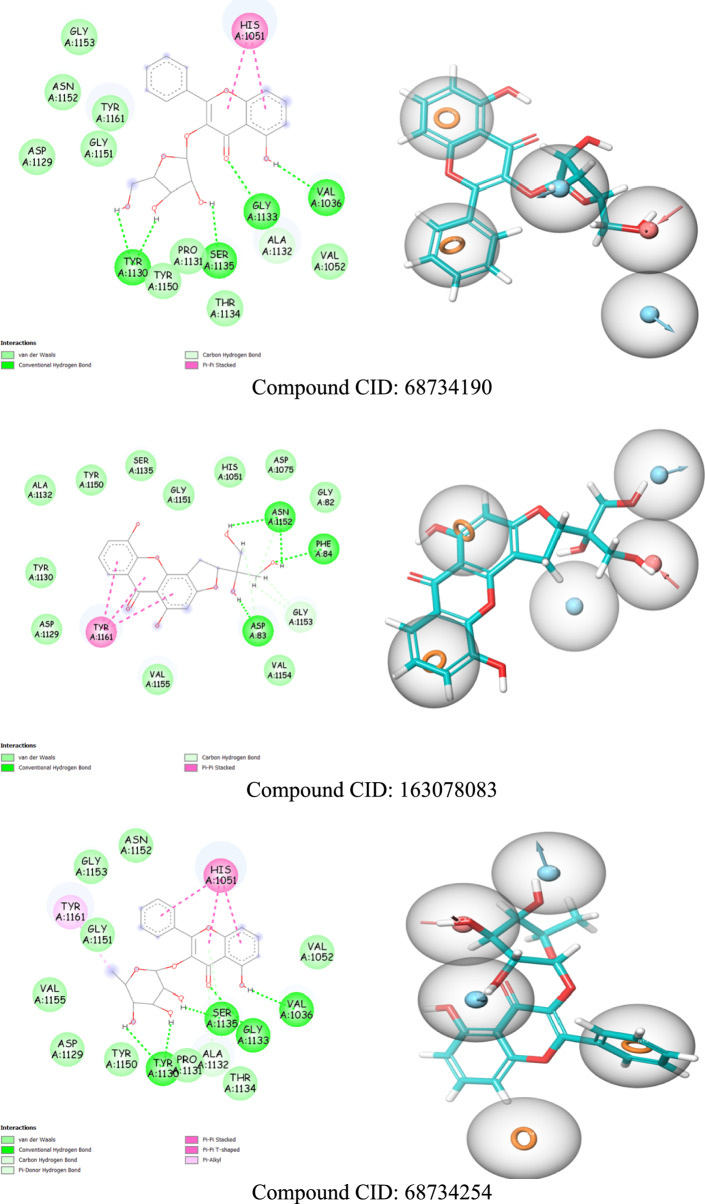

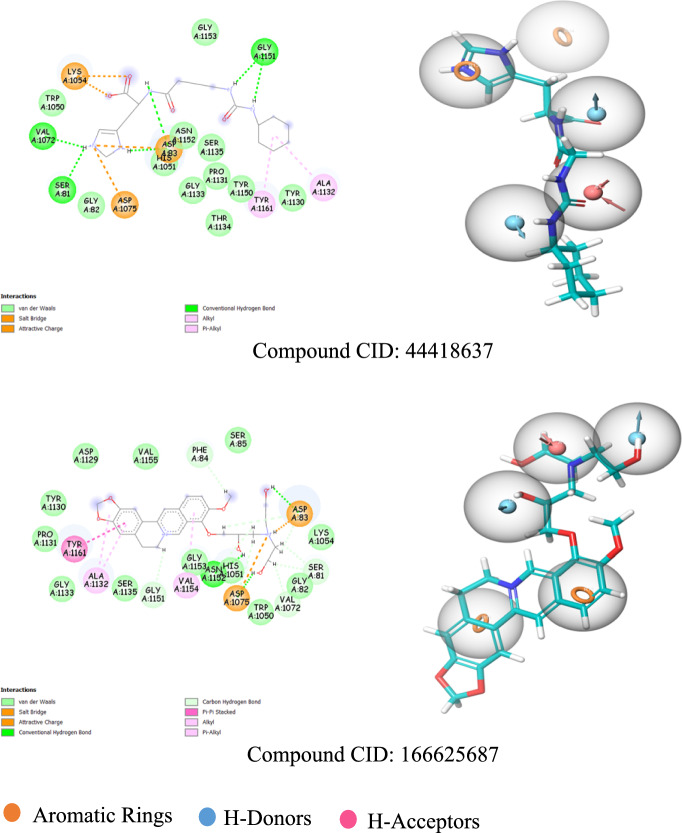
**a** The binding interactions map (2D). **b** The e-pharmacophore matches ligands with the ZIKV NS2B-NS3 active site

The e-pharmacophore matches and binding interactions of the ligands with the ZIKV NS2B-NS3 protease are illustrated in Fig. 3.

Three types of bonds were formed through the interactions of the ligands with the selected binding site: hydrogen bonds, electrostatic bonds, and hydrophobic bonds. These interactions are summarized in Table A1. Similar hydrophobic interactions involving π-interactions at the chromene rings of all flavonoid ligands with the His1051 and Tyr1161 amino acids were observed. These interactions underscore the significance of the aromatic ring features mapped in our e-pharmacophore model for binding to the ZIKV NS2B-NS3 cavity.

Another hydrophobic bond was also noted between Ala1132 and Val1072 with the cyclohexane and imidazole or benzodioxole rings of the CID 44418637 and CID 16662587 ligands, respectively. These compounds also formed electrostatic interactions characterized as salt bridges, where the acceptor and donor atoms are fully charged, facilitating hydrogen bonds between the nitrogen atoms and the Asp1075, Asp83, and Lys1054 amino acids.

A thorough examination of the docked ligands revealed that Asp83, Asp1075, Ser1135, Gly1151, Gly1133, Tyr1130, Asn1152, Phe84, His1051, and Tyr1161 play crucial roles in hydrogen bonding interactions with the alcohol and ketone functionalities of the compounds, acting as hydrogen bond acceptors and donors. This highlights the importance of these donor and acceptor hydrogen bond features, which align with our e-pharmacophore model for the binding and stability of compounds at the active site of the ZIKV NS2B-NS3 protease. Notably, these amino acids in the NS2B-NS3 protease binding site have also been identified in recent research [23, 29].

The hydrogen bonding distances ranged from 1.6 Å to 3.1 Å, which is considered optimal, as the typical distance for hydrogen bonding is around 3 Å [45]. Based on these interaction analyses and docking results, our natural derivative compounds exhibit strong binding affinity with the protease and are regarded as more promising inhibitors of the NS2B-NS3 protease than the boronate inhibitor. Further ADME studies will provide additional confirmation of their potential.

### ADME Prediction

Respectable ADME (Absorption, Distribution, Metabolism, and Excretion) qualities are essential for the successful development of a drug, in addition to strong efficacy [46]. This study assessed the highest-scoring compounds from docking for ADME properties analysis using the SwissADME server, with all results listed in Table A2.

In our analysis, all compounds adhered to Lipinski’s rules (molecular weight < 500, H-bond donors < 5, H-bond acceptors < 10, log P < 5) [47] and Veber’s criteria (rotatable bonds < 10, TPSA < 140 Å^2^) [48], except for three compounds that had one violation. Specifically, CID 44418637 had 12 rotatable bonds, while the TPSA values for CID 163078083 and CID 58178603 were 140.59 Å^2^, exceeding the threshold of 140 Å^2^, albeit only slightly.

Solubility is also crucial for the pharmacological response of orally administered drugs. Based on the ESOL log S model implemented by John S. Delaney (2004), most of the analyzed compounds exhibited solubility, facilitating their excretion through the renal pathway. The compounds demonstrated acceptable absorption due to their lipophilicity, with log P values ranging from 0.75 to 2.23—well below the threshold of five. This suggests high gastrointestinal absorption and limited permeability across the blood–brain barrier (BBB), as indicated by the BOILED-Egg model shown in Fig. A3. These results further confirm the compounds' ability to cross biological membranes to reach their specific target sites, supported by their bioavailability values, which indicate the extent to which a substance becomes accessible to its intended biological site [49]. The acceptable probability value for bioavailability is 0.55 [50], and all compounds met this criterion.

Furthermore, cytochrome P450 (CYP) enzymes play a vital role in drug detoxification, and the ability of a drug to inhibit certain CYP enzymes is critical to consider. In this study, all compounds were metabolized easily by the digestive system due to their lack of inhibition on CYP1A2, CYP2C19, CYP2C9, CYP2D6, and CYP3A4, with two exceptions. CID 166625687 exhibited inhibitory action on CYP3A4, which metabolizes more than 30% of drugs [51], while CID 42605183 inhibited CYP2D6, an isoenzyme that metabolizes around 25% of known drugs [52]. Additionally, most compounds showed potential for efflux from the central nervous system via P-glycoprotein substrates, which is significant given the Zika virus's preference for placental tissue. P-glycoprotein is a prominent ABC transporter found in the apical layer of syncytiotrophoblasts [53], suggesting that the predicted compounds could help mitigate microcephaly syndrome in pregnant women caused by the Zika virus.

Notably, the synthetic accessibility (SA) score is a crucial metric in drug discovery, where a score of 1 indicates simple synthesis, and a score of 10 indicates complex synthesis [54, 55]. In our analysis, all compounds had an SA score of less than 5, indicating their synthesis would be relatively straightforward. Overall, the ADME profiles of the selected compounds were generally preferable compared to the boronate inhibitor (CID 137348520), except for CID 166625687 and CID 42605183, which were excluded from toxicity predictions due to their inhibitory interactions with CYP3A4 and CYP2D6. The radar plots for each compound are illustrated in Fig. A4.

**Fig. 4:**
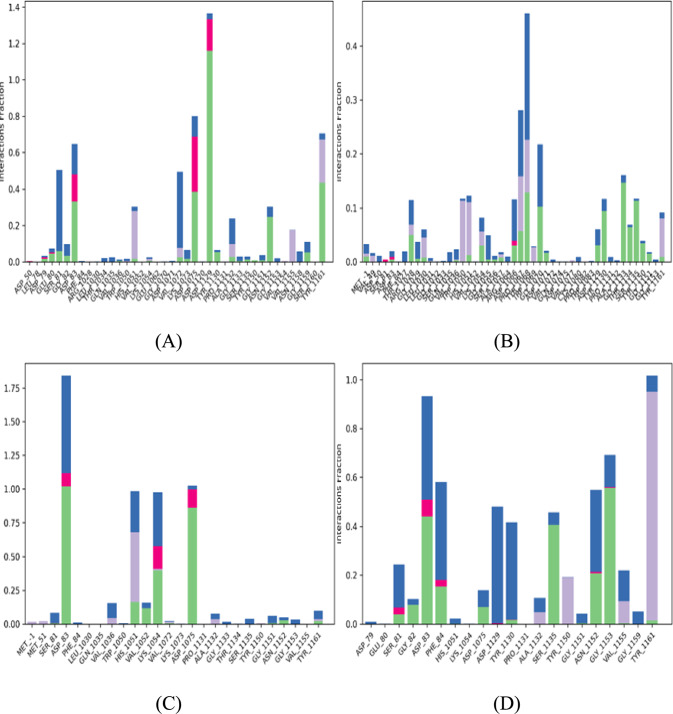
Comparison of Protein-compound contacts histogram for different compounds. **a** Reference compound. **b** Compound 68734190. **c** Compound 44418637. **d** Compound 163078083

### Toxicity and Bioactivity Prediction

The top leads with favourable ADME properties underwent toxicity analysis using ProTox-ll. The results are summarized in Table A3. All compounds were deemed safe except for CID 68734254, which exhibited carcinogenicity and immunotoxicity, and CID 58178603, which showed mutagenicity and cytotoxicity. The median lethal dose (LD50) was also assessed, with three compounds—CID 68734254, CID 68734190, and CID 58178603—having an LD50 of 5000 mg/kg, placing them in toxicity class V. CID 44418673 had a lower LD50 of 3000 mg/kg but also fell into class V. The remaining compounds, including CID 166479806 and CID 1630780863, belonged to class IV, with LD50 values of 1000 and 562 mg/kg, respectively.

Flavonoids are recognized for their significance in treating viral infections and preventing chronic diseases, and they are generally considered safe. Additionally, peptides are interesting due to their nontoxicity, selective bioactivity, and effectiveness [56, 57]. Among our non-toxic compounds, most are flavonoids, with one being a dipeptide (CID 166479806, CID 44418637, CID 163078083, CID 68734190). Several studies have indicated that flavonoids serve as effective antiviral agents during various phases of viral infection, particularly in targeting replication proteins [58]. Other investigations have demonstrated that dipeptides possess antiviral activity against HIV-1 protease [59, 60].

Consequently, the top leads were subjected to bioactivity prediction as a supportive analysis, showing significant antiviral activity as evaluated on the PASSonline website. All compounds were predicted to target the replicase polyprotein 1ab and the human immunodeficiency virus type 2 integrase, with moderate confidence scores ranging from 0.3649 to 0.9781. Notably, CID 44418637 displayed different targeting, interacting with the Dengue virus type 2 NS3 protein and the HIV-2 pol protein, with confidence scores of 0.1061 and 0.0572, respectively. The bioactivity prediction results are illustrated in Table A4.

These targets are crucial for viral replication and maturation, facilitating the insertion of the viral genetic material into the host cell DNA. In particular, the replicase polyprotein 1ab plays a vital role in transcription and viral replication [61], supporting our study’s relevance to the role of the NS2B-NS3 protease in viral replication.

After a thorough analysis, three compounds (CID 44418637, CID 163078083, and CID 68734190) were selected for molecular dynamics simulations. At the same time, CID 166479806 was excluded due to its higher molecular weight compared to the chosen compounds.

### Molecular Dynamic

To further investigate binding stability, a molecular dynamics (MD) study was conducted for 100 ns using the Desmond package on the top three compounds—CID 44418637, CID 163078083, and CID 68734190—each with favourable ADMET profiles, alongside the reference compound. The analysis included Protein-Compound Contacts Timeline, Secondary Structure Timeline, Protein-Compound Contacts Histogram, RMSD (Root Mean Square Deviation), and RMSF (Root Mean Square Fluctuation) values, providing insights into the binding stability of the docked complexes.

The RMSD values reflected the behaviour of atomic positions, showing notably larger variations in regions corresponding to protein–ligand interactions. Smaller RMSD fluctuations indicate more stable ligand binding.

The timeline in Fig. A5 summarizes the intermolecular contacts between the protein and ligand during the simulation. The upper panel tracks the total number of specific interactions (hydrogen bonds, ionic bonds, hydrophobic interactions, and water bridges) formed at each time point. The lower panel highlights the individual protein residues involved in these interactions throughout the trajectory. Residues that establish multiple specific contacts with the ligand are represented by darker shades of orange, as indicated by the scale on the right of the plot.

**Fig. 5:**
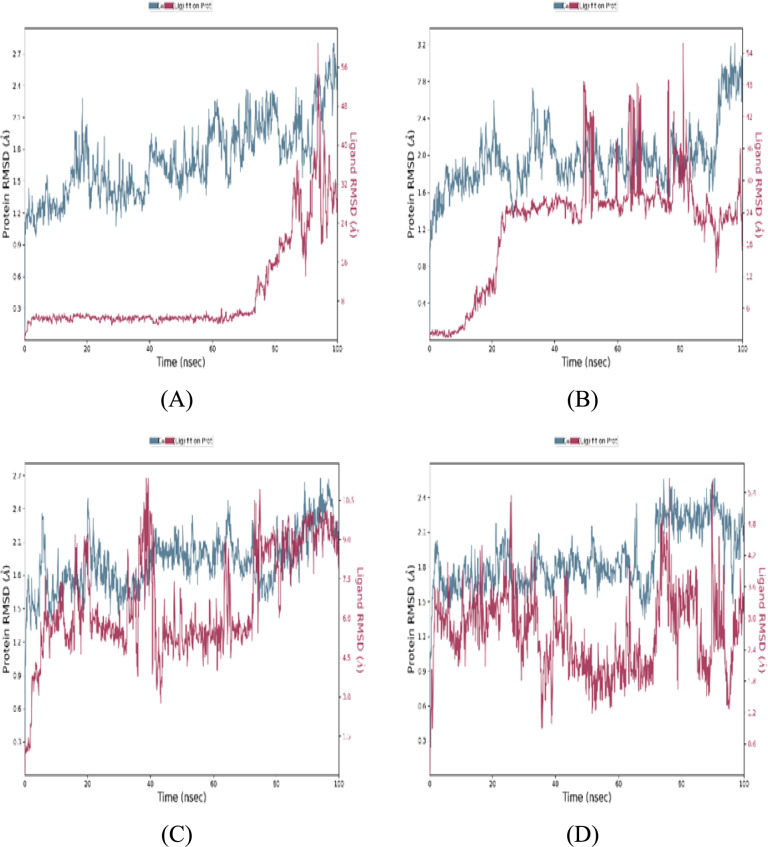
Comparison of protein-compound RMSD for different compounds. **a** Reference compound. **b** Compound 68734190.** c ** Compound 44418637.** d** Compound 163078083

The reference compound maintained relatively stable interactions with the protein until 65 ns, demonstrating a consistent number of protein–ligand contacts throughout the simulation.

Interestingly, the protein–ligand interactions began to fluctuate after 65 ns. Compound CID 68734190 demonstrated varying interactions with the protein, experiencing periods of both low and high contact numbers, suggesting that it may exhibit dynamic binding behaviour. In contrast, compound CID 44418637 maintained a pattern similar to the reference compound, characterized by stable interactions and a consistent number of protein–ligand contacts throughout the simulation duration.

Compound CID 163078083 displayed a distinct interaction pattern compared to the other compounds, featuring an initial interaction spike followed by a gradual decline and a subsequent spike. This behaviour indicates that CID 163078083 possesses a unique chemical architecture, allowing it to bind to the target protein differently.

The stability of protein secondary structure elements (SSEs), such as alpha-helices and beta-strands, was closely monitored throughout the MD simulation trajectories of all compound-protein complexes. Figure A6 illustrates the distribution of SSEs across the protein structure based on residue index. The upper plot summarizes the SSE composition for each trajectory frame throughout the simulation, while the lower plot tracks the SSE assignments for each residue over time.

**Fig. 6:**
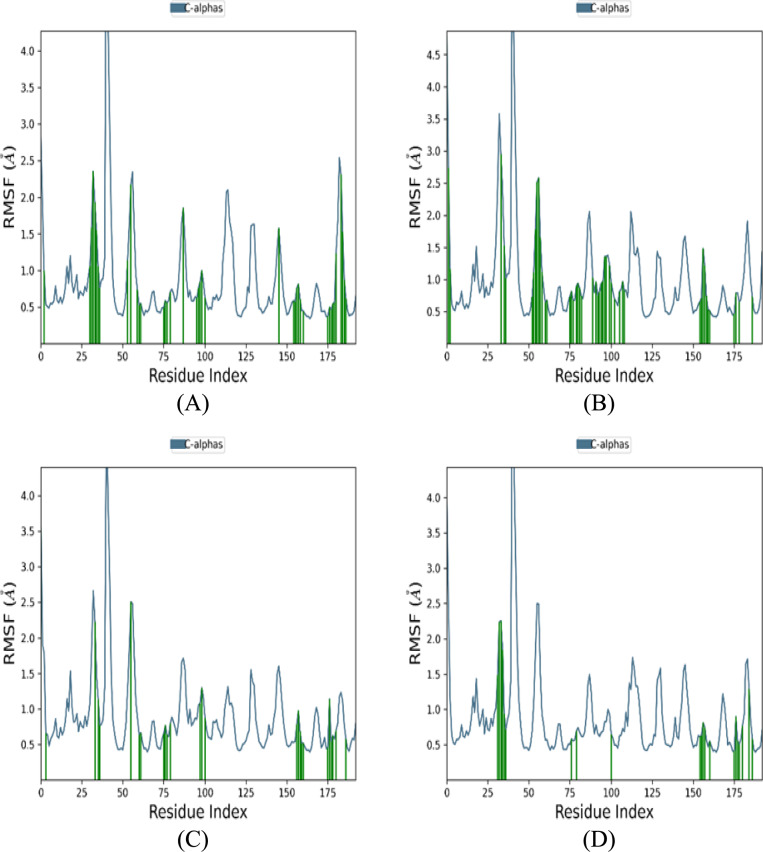
Comparison of Protein-RMSF for different compounds.** a** Reference compound. **b** Compound 68734190. **c** Compound 44418637.** d** Compound 163078083

Analysis of the secondary structure elements during the 100 ns molecular dynamics simulations revealed that the protein domains exhibited remarkable stability, particularly for extended conformations (depicted in sky blue). However, a specific region encompassing residues 160–175 displayed persistent fluctuations and alterations in their SSE assignments across all compounds, including the reference compound.

The analysis indicates that the compound CID 44418637–complex exhibits the most stable secondary structure compared to CID 163078083. Protein complexes with CID 68734190 and CID 44418637 occupy intermediate positions regarding secondary structure stability. A comprehensive assessment of SSE dynamics revealed that the identified compounds exhibit superior stability compared to the reference compound complexes, which exhibited the most dynamic behaviour concerning SSE formation and disruption.

Figure 4 presents a comparative analysis of protein-compound interactions using histograms for different compounds. These histograms depict the distribution of protein-compound contacts, classified into four key categories: hydrogen bonds, hydrophobic interactions, ionic interactions, and water bridges. Each category includes several subcategories, which can be visualized and analyzed within the "Simulation Interactions Diagram" panel.

Figure 4 visualizes the average duration of different interaction types, such as hydrogen bonds (H-bonds), during the simulation. For example, a value of 0.75 for H-bonds indicates that these interactions persist for an average of 75% of the simulation time. Values above 1.0 are also possible, as some protein residues can form multiple H-bonds with the ligand, highlighting their significance in binding.

Hydrogen bonds play a critical role in drug design because they influence specificity, metabolism, and absorption. Within protein–drug interactions, these bonds can be categorized into four types based on their origin: backbone and side-chain acceptors/donors. Researchers employ specific geometric criteria to identify protein–ligand interactions, including a 2.5 Å distance and defined angle constraints for hydrogen bonds. Hydrophobic contacts are divided into three categories (π–cation, π–π, and non-specific) based on distance and interaction types between protein and ligand groups. Recently, π–cation interactions, typically involving hydrophobic amino acids and aromatic/aliphatic ligand groups, have been included within these criteria.

Beyond hydrogen bonds, identifying protein–ligand interactions also involves examining ionic interactions, where oppositely charged atoms attract each other within 3.7 Å. The involvement of the protein backbone or side chains can further categorize these interactions. Additionally, protein–metal–ligand interactions are considered, where metal ions are coordinated within 3.4 Å of the protein and ligand's heavy atoms (excluding carbon).

Water bridges, mediated by water molecules, represent hydrogen-bonded interactions with slightly relaxed criteria: a distance of 2.8 Å between donor and acceptor atoms, a minimum donor angle of 110°, and a minimum acceptor angle of 90°. Understanding these diverse interaction types and their specific geometric criteria is crucial for comprehensively analysing protein–ligand interactions.

Analysis indicates that the reference compound exhibits more interactions; however, these interactions are not as stable compared to the hydrogen bonding interactions demonstrated by the identified compounds.

Figure 5 compares the RMSD (Root Mean Square Deviation) evolution of the reference compound and the three top-ranked compounds in the complex with the protein. The left Y-axis represents the protein RMSD, calculated by aligning all protein frames to the reference frame backbone and measuring the RMSD based on the selected atoms. The right Y-axis represents the ligand RMSD (denoted as 'Lig fit Prot'), which indicates the stability of the ligand concerning the protein and its binding pocket. The observed RMSD changes fall within the acceptable range for small, globular proteins (1–3 Å).

The RMSD analysis indicates that all four compounds exhibit mixed equilibrated behaviour, with RMSD fluctuations stabilizing around a fixed value throughout the simulation. The ligand RMSD for the reference compound remains relatively constant until 85 ns, after which it exhibits some fluctuations; however, these variations remain within the acceptable range throughout the simulation, suggesting that the ligand is tightly bound to the protein's binding site.

In contrast, the ligand RMSD for compounds CID 68734190 and CID 44418637 shows a steady increase, indicating that these compounds may not be as stable within the protein's binding pocket. Compound CID 163078083 displays a more pronounced fluctuation in ligand RMSD, suggesting a dynamic interaction between the ligand and the protein.

These RMSD results offer valuable insights into the structural dynamics of the protein–ligand complexes, suggesting that the different chemical structures of the compounds contribute to variations in binding stability.

Figure 6 illustrates the RMSF (Root Mean Square Fluctuation) profiles of the reference compound and the three top-ranked compounds in the complex with the protein. RMSF measures local flexibility along the protein chain and is calculated as the average root-mean-square displacement of atoms in a residue from their average position over the entire simulation trajectory. Regions with high RMSF values correspond to protein areas exhibiting significant fluctuations. Typically, proteins' N-terminal and C-terminal tails demonstrate higher RMSF values, indicating greater flexibility compared to the more rigid core regions. Secondary structure elements, such as alpha helices and beta strands, are generally more stable and exhibit lower RMSF values than loop regions, characterized by greater flexibility and mobility.

The RMSF profiles for the reference compound and the compounds CID 68734190, CID 44418637, and CID 163078083 exhibited variations in residue-level flexibility. The reference compound shows a relatively smooth RMSF profile, suggesting a balanced distribution of flexibility throughout the protein. Compounds CID 68734190 and the reference compound demonstrate increased RMSF values in specific regions, indicating localized variations in flexibility. In contrast, compounds CID 44418637 and CID 163078083 exhibit less pronounced overall RMSF, suggesting a more stable complex when binding to the protein structure.

## MM/GBSA Analysis

Post-MD MM/GBSA calculations were performed on the three top hit compounds and the Reference Compound to validate the molecular dynamics simulations further. Using the Prime module in Maestro, these calculations assessed the binding free energy (ΔE) of each compound to the ZIKV NS2B-NS3 protease. As shown in Table 2, the MM/GBSA analysis results were consistent with the observations of the MD simulation. Compound 68734190 exhibited the most favourable binding affinity with a ΔE of − 50.192 kcal/mol, followed closely by Compound 163078083 with a ΔE of − 49.947 kcal/mol. These compounds demonstrated significantly stronger binding than the Reference Compound, which had an ΔE of − 38.347 kcal/mol. Compound 44418637 displayed the weakest binding affinity among the analyzed compounds, with an ΔE of − 26.909 kcal/mol. The superior binding affinities of Compounds 68,734,190 and 163,078,083 suggest they are promising candidates as potential inhibitors of the ZIKV NS2B-NS3 protease and warrant further investigation.

**Table 2:** MM/GBSA-Calculated Binding Free Energies (ΔE) of Compounds to the ZIKV NS2B-NS3 Protease

Compound	Overall binding free energy ΔE	The total energy of the complex EC	The total energy of the receptor ER	The total energy of the ligand EL
68734190	− 50.192	− 5422.452	− 5255.723	− 116.537
44418637	− 26.909	− 5355.775	− 5272.989	− 55.877
163078083	− 49.947	− 5386.213	− 5289.493	− 46.773
Reference	− 38.347	− 6450.188	− 6355.652	− 56.189

## Discussion

The NS2B-NS3 enzyme complex is critical for viral replication within host cells and plays a role in helping viruses evade innate defense mechanisms [14]. Therefore, inactivating this enzyme presents an intriguing target for developing antiviral treatments against the virus. Using computational approaches, our investigation aimed to predict and identify new natural inhibitors of the Zika virus NS2B-NS3 protease. An e-pharmacophore model was employed as the first stage for the virtual screening of our natural derivative compounds sourced from the PubChem database to achieve this goal. The generated model hypothesis comprised five points (ADDRR) to screen the entire database. Following this, molecular docking was utilized as an in-silico tool to predict the binding poses of small molecules at the receptor's active site and assess their affinity by ranking them based on score values. Two techniques were employed for this screening: SP docking for the larger pool of compounds, followed by XP docking, which yielded eight hits with the best scores—CID 166479806, CID 166625687, CID 68734254, CID 44418637, CID 163078083, CID 42605183, CID 68734190, and CID 58178603—ranging from − 7.110 to − 9.166 kcal/mol.

In contrast, the score for the boronate inhibitor (the reference ligand) was − 5.492 kcal/mol, indicating that our compounds demonstrate significantly better affinity for the binding site. Regarding interactions, important hydrogen bonds were observed between the selected hits and key amino acid residues of the NS2B-NS3 active site, including Asp83, Asp1075, Ser1135, Phe84, His1051, Tyr1130, Gly1151, Gly1133, and Asn1152. Notably, these amino acids in the NS2B-NS3 protease binding site have also been highlighted in recent studies [23, 29].

These results underscore the potential of the selected hits to inhibit the biological activity of the target NS2B-NS3 ZIKV protease. In terms of pharmacokinetic characteristics, all compounds exhibited adequate absorption, as evidenced by their logP values below five, which suggests strong gastrointestinal absorption. This indicates their permeability to traverse biological membranes to target specific sites, corroborated by their bioavailability value 0.55. When considering the inhibition of cytochrome P450 (CYP) enzymes, our compounds metabolized efficiently through the digestive system due to their lack of inhibitory effects on CYP enzymes, except for CID 166625687 and CID 42605183, which exhibited inhibition against two essential enzymes, CYP3A4 and CYP2D6, respectively. Additionally, our compounds are likely easily eliminated through renal excretion due to their high solubility.

The pharmacokinetic analysis indicated that our suggested hits possess a favourable ADME profile, except for the two compounds above. After a series of screening examinations, six leads were selected for toxicity prediction. Since our compounds are natural derivatives, primarily flavonoids, their toxicity is generally low and considered safe, with CID 68734254 and CID 58178603 being the exceptions that showed certain toxicity. Furthermore, multiple studies indicate that flavonoids and peptides are significant as antiviral agents due to their efficacy at various stages of viral infection, including replication processes. For this reason, the best leads underwent bioactivity prediction as a supplementary analysis, demonstrating significant activity with the replicase polyprotein 1ab. Ultimately, three leads were selected for molecular dynamics simulations, showing stable complexes with the NS2B-NS3 protease throughout 100 ns of simulation. Based on our findings, these leads could be promising candidates for potential antiviral agents targeting the ZIKV NS2B-NS3 protease.

In addition to the findings presented, it is crucial to acknowledge the limitations inherent in computational modelling, including the accuracy of predictions and the necessity for experimental validation. While our study identified promising natural compounds with potential inhibitory effects on the NS2B-NS3 protease, computational methods cannot fully substitute for empirical data. Therefore, we recommend that future research focus on the experimental assessment of these compounds to confirm their binding efficacy and therapeutic potential. This approach will validate our computational results and provide insights into the biological relevance of the identified inhibitors, ensuring a more robust understanding of their applicability in drug development.

## Conclusions

Zika virus (ZIKV) poses a significant global public health challenge, as there are currently no effective vaccines or treatments available. However, our study, conducted using in silico methods, has identified and predicted novel natural inhibitors of the NS2B-NS3 ZIKV protease. Specifically, this study identified three natural compounds (CID 68734190, CID 44418637, and CID 163078083) as potential inhibitors of the NS2B-NS3 ZIKV protease, a crucial target for antiviral drug development. Molecular docking simulations and MM/GBSA calculations predicted favourable binding affinities, particularly for compounds CID 68734190 and CID163078083. Molecular dynamics simulations also demonstrated stable interactions between these compounds and the protease. These findings highlight the potential of these natural compounds as promising leads for the development of effective treatments for ZIKV infections. However, further experimental validation through in vitro and in vivo studies is crucial to confirm their biological activity and therapeutic efficacy.

## Supplementary Information

Below is the link to the electronic supplementary material.

Supplementary file1 (DOCX 2055 KB)

Supplementary file2 (DOCX 166 KB)

## Data Availability

The datasets used in the manuscript are publicly available from the repositories below: (1) Repository Name: RCSB Protein Data Bank; Deposited Date: 2016–06-18; Released Date 2016–07-06; by source author(s): Lei, J., Hansen, G., Zhang, L.L., Hilgenfeld, R. Accession Number: 10.2210/pdb5LC0/pdb; Macromolecular structure:5LC0[link to the repository: https://www.rcsb.org/structure/5LC0] and originally deposited from article, 10.1126/science.aag2419, and (2) Repository Name: PubChem database [link to the repository: https://pubchem.ncbi.nlm.nih.gov/] and (3) other datasets generated during the current study can be made available from the corresponding author upon reasonable request.
