# Supplementary material for: Identification and Evaluation of Natural Compounds as Potential Inhibitors of NS2B-NS3 Zika Virus Protease: A Computational Approach
Source: Mol Biotechnol. 2024 Dec 28;67(12):4632–50. doi: 10.1007/s12033-024-01357-6 (PMC12634716; doi:10.1007/s12033-024-01357-6)
Supplement: Supplementary file 1 — Supplementary file1 (DOCX 2055 KB) [file 12033_2024_1357_MOESM1_ESM.docx]

**Appendix A**

**Identification and Evaluation of Naturel Compounds as Potential inhibitors of NS2B-NS3 Zika Virus protease: A Computational Approach**

**Nada Anede ^1^, Mebarka Ouassaf ^1,^ *, Kannan RR Rengasamy ^2,3^, Shafi Ullah Khan ^4,5^, and Bader Y. Alhatlani ^6,^ ***

^1^ Group of Computational and Medicinal Chemistry, LMCE Laboratory, University of Biskra, Biskra, Algeria; [nada.anede@univ-biskra.dz](mailto:nada.anede@univ-biskra.dz); [nouassaf@univ-biskra.dz](mailto:nouassaf@univ-biskra.dz)

^2^ Laboratory of Natural Products and Medicinal Chemistry (LNPMC), Saveetha Medical College and Hospital, Saveetha Institute of Medical and Technical Sciences (SIMATS), Thandalam, Chennai, 602105 India. [Ragupathi.Rengasamy@nwu.ac.za](mailto:Ragupathi.Rengasamy@nwu.ac.za)

^3^ Centre of Excellence for Pharmaceutical Sciences, North-West University, Potchefstroom, 2520, South Africa

^4^ UNICAEN, Inserm U1086 ANTICIPE (Interdisciplinary Research Unit for Cancer Prevention and Treatment), Normandie Univ, Caen, France; [shafiullahpharmd@gmail.com](mailto:shafiullahpharmd@gmail.com)

^5^ Cancer Centre François Baclesse, UNICANCER, Caen, France ; [shafiullahpharmd@gmail.com](mailto:shafiullahpharmd@gmail.com)

^6^ Unit of Scientific Research, Applied College, Qassim University, Buraydah 52571, Saudi Arabia; [balhatlani@qu.edu.sa](mailto:balhatlani@qu.edu.sa)

***** Correspondence: [balhatlani@qu.edu.sa](mailto:balhatlani@qu.edu.sa) (B.Y.A.); [nouassaf@univ-biskra.dz](mailto:nouassaf@univ-biskra.dz) (M.O.); [Ragupathi.Rengasamy@nwu.ac.za](mailto:Ragupathi.Rengasamy@nwu.ac.za) (KRRR).


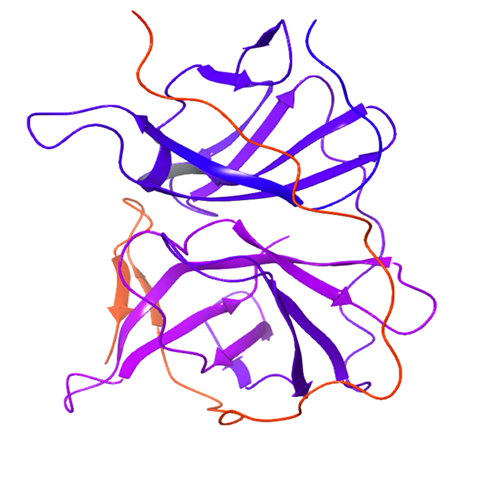


**Fig A1.** The three-dimensional structure of the prepared monomer NS2B-NS3 protease (PDB ID: 5LC0).

| 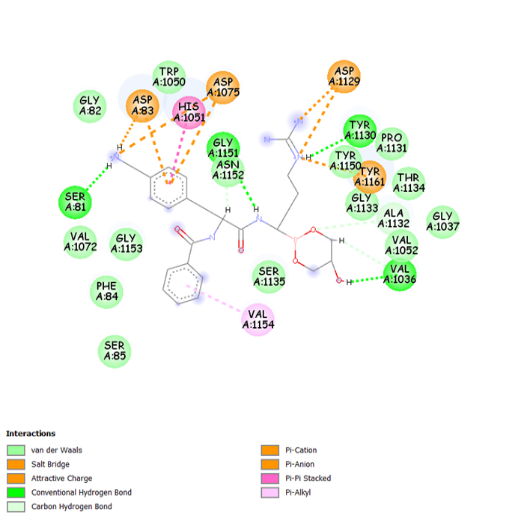 | 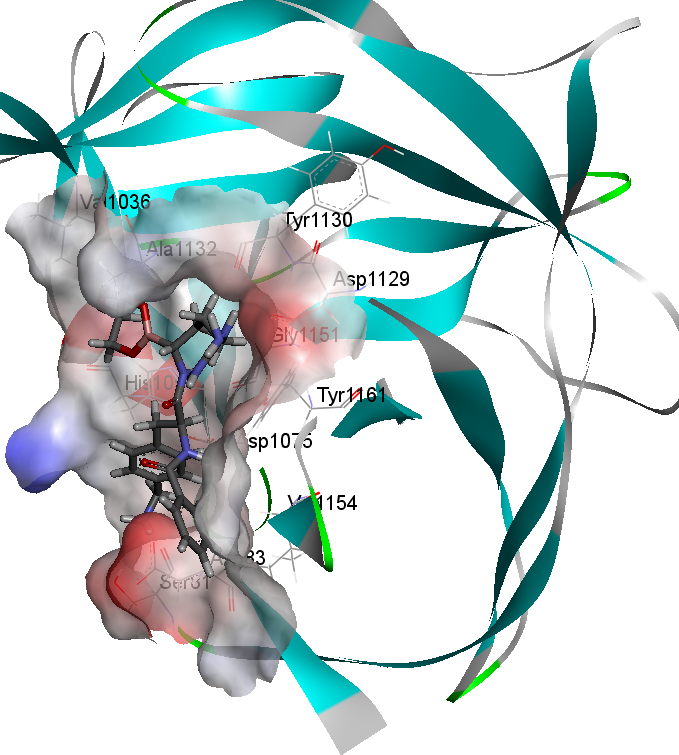 |
| --- | --- |

**Fig A2.** The important residues contribute to substrate binding of the active site visualizing by Discoverystudio.


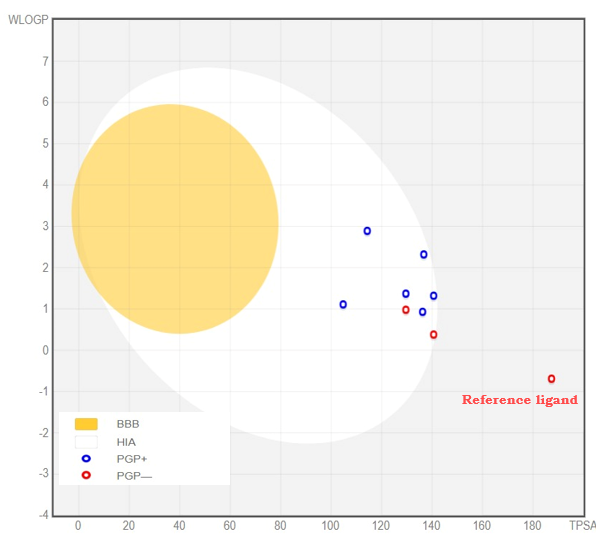


**Fig A3.** The BOILED-Egg model of the top docked ligands.

| 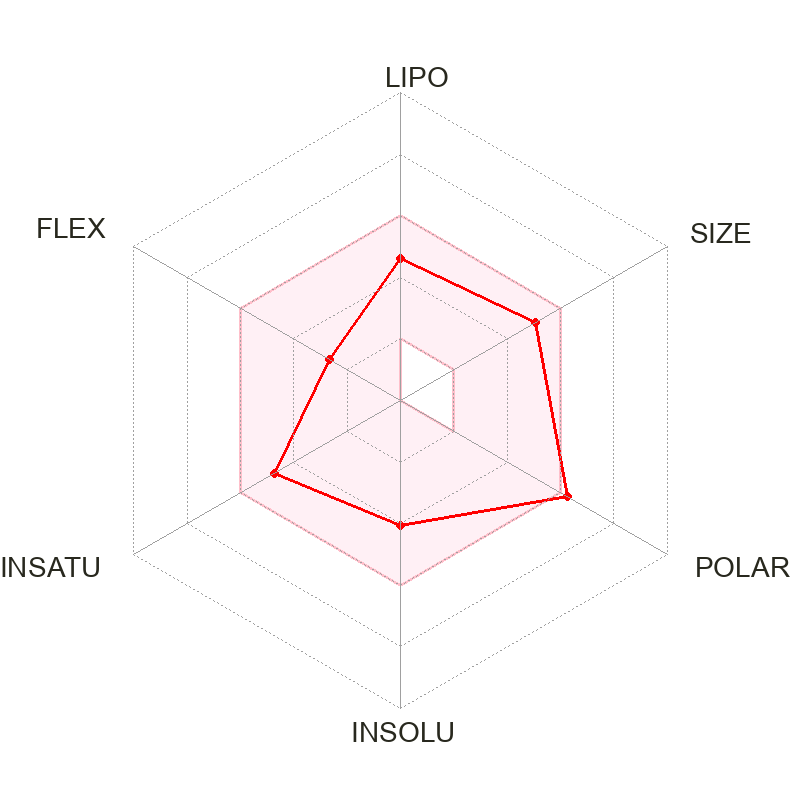 | 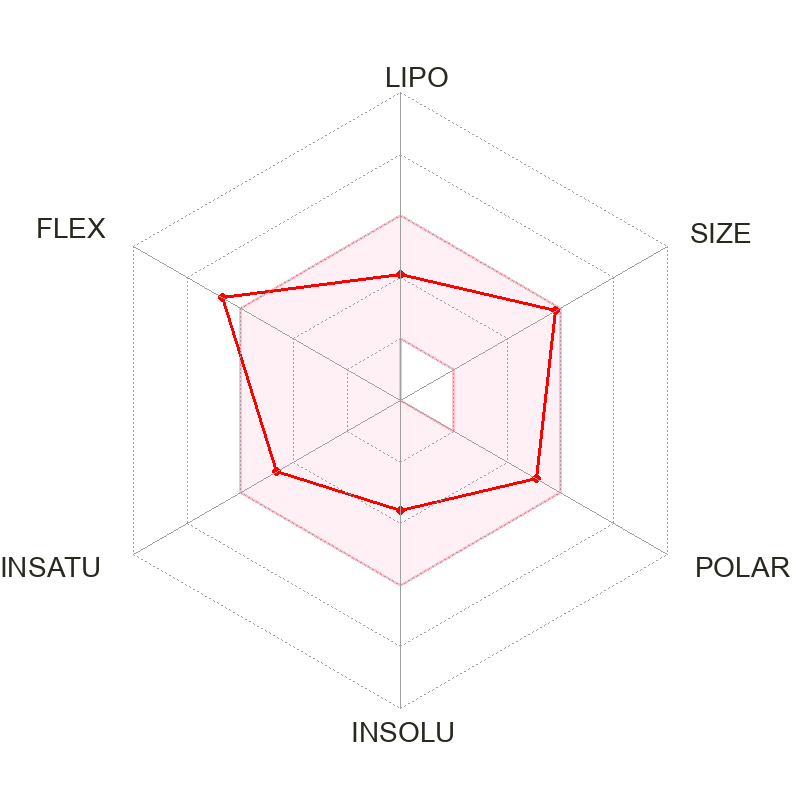 |
| --- | --- |
| CID 166479806 | CID 166625687 |
| 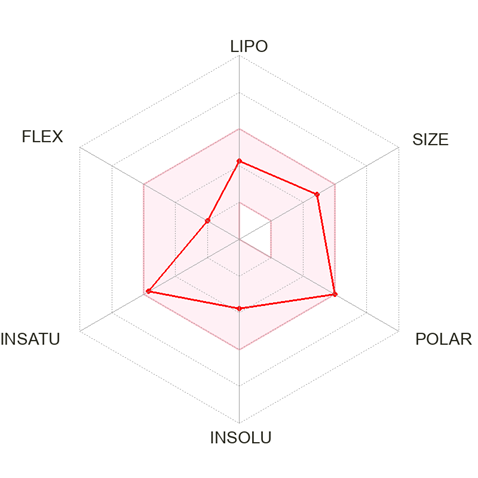 | 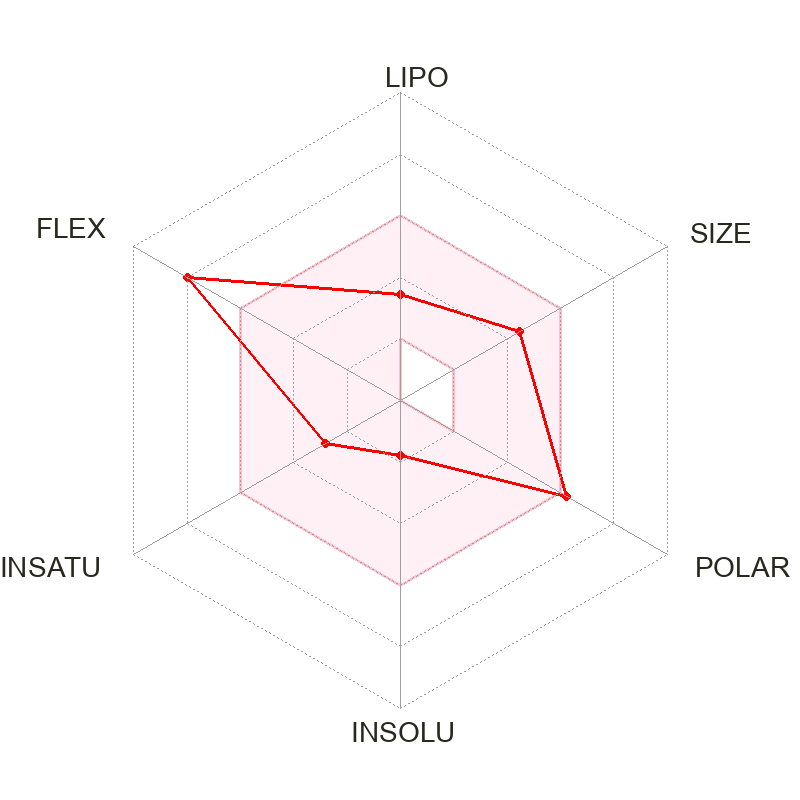 |
| CID 68734254 | CID 44418637 |
| 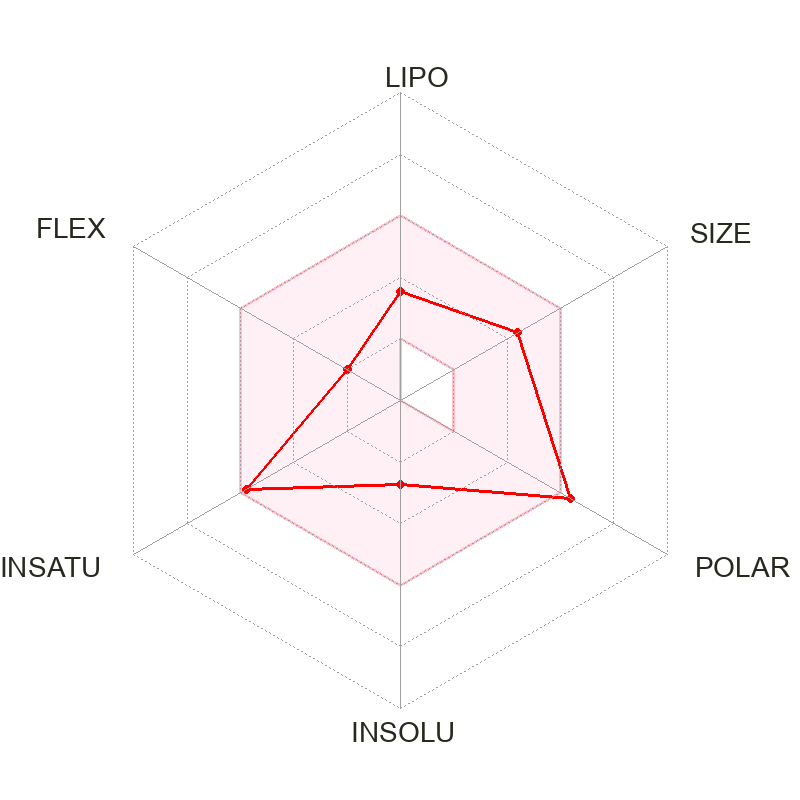 | 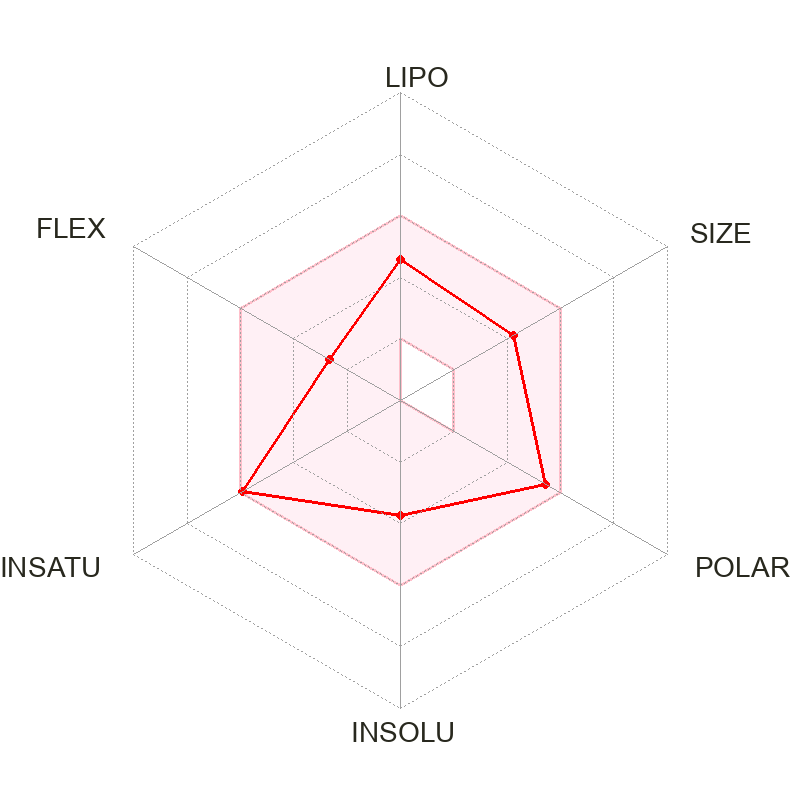 |
| CID 163078083 | CID 42605183 |
| 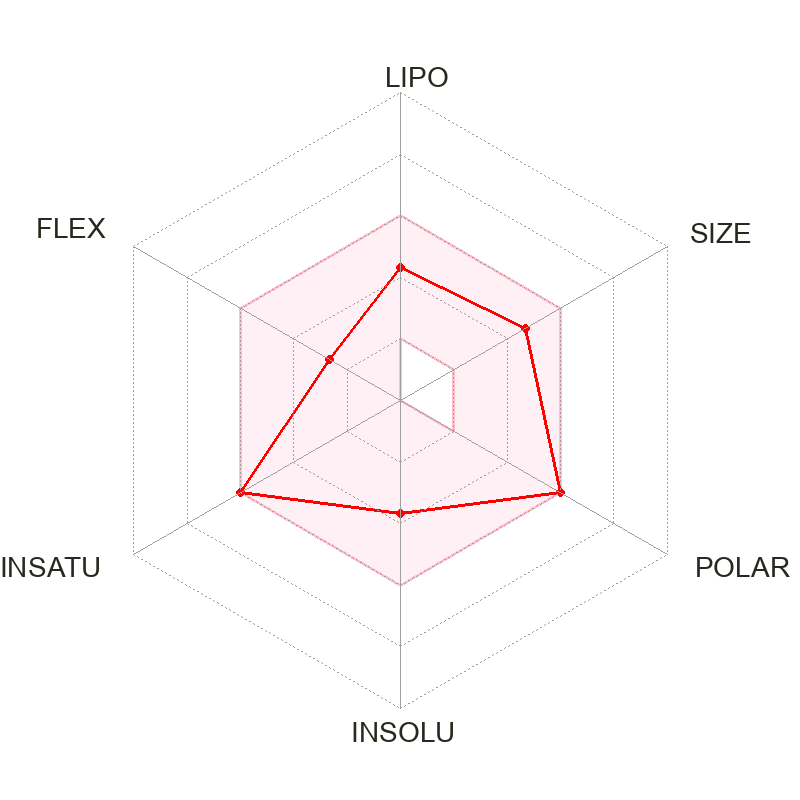 | 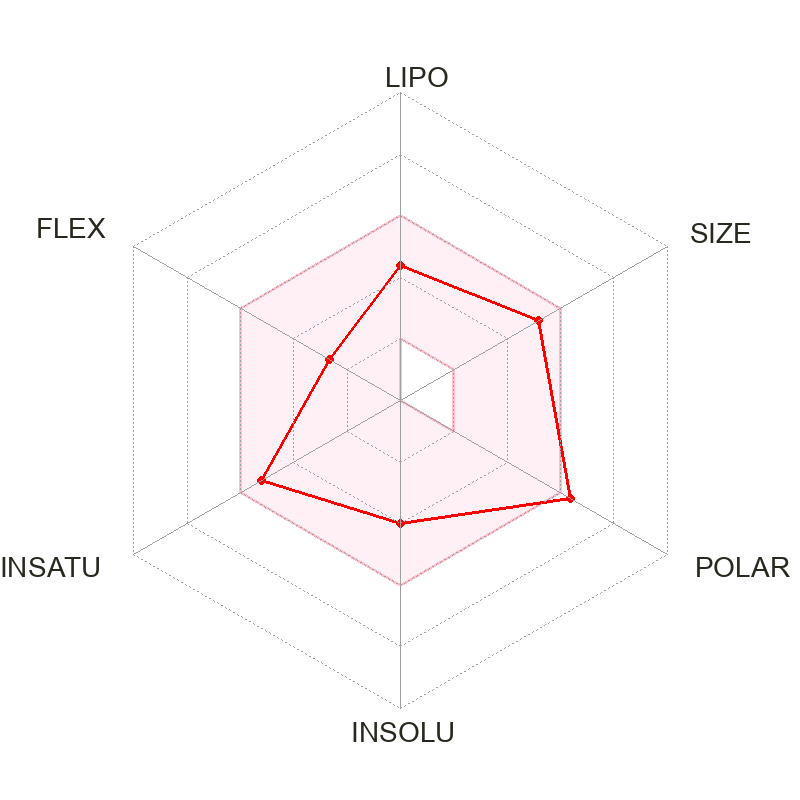 |
| CID 68734190 | CID 58178603 |
| 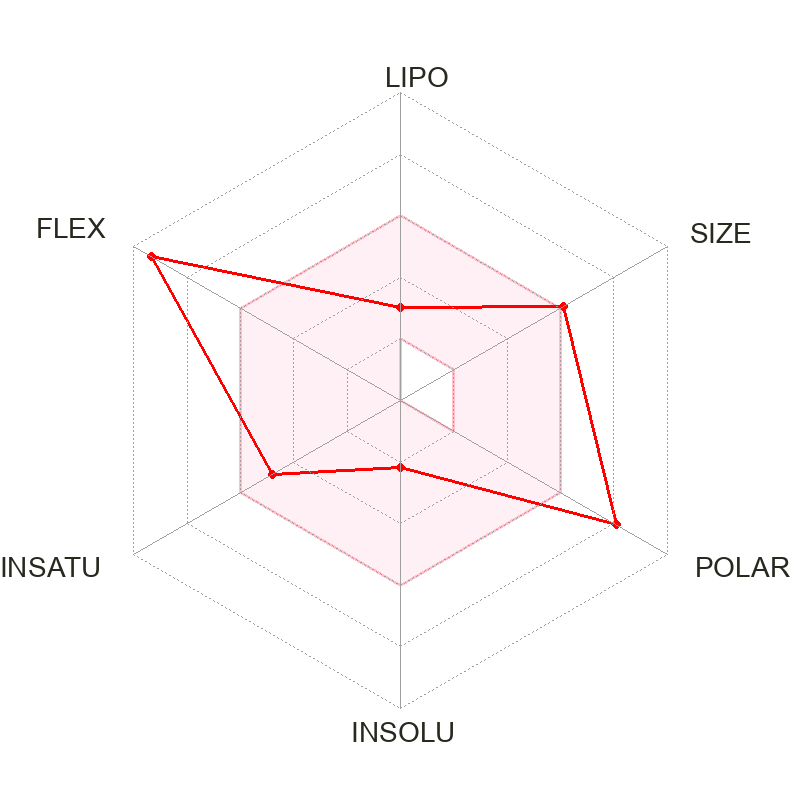 | |
| Reference ligand CID 137348520 | |

**Fig A4.** The radar of the ADME analysis of the top docked ligands.

| 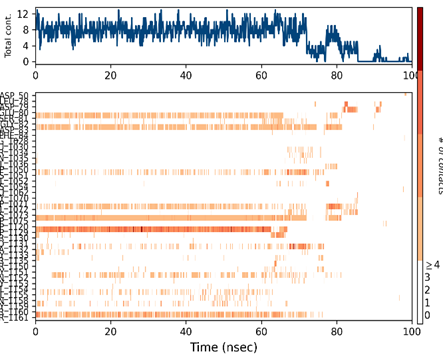 | 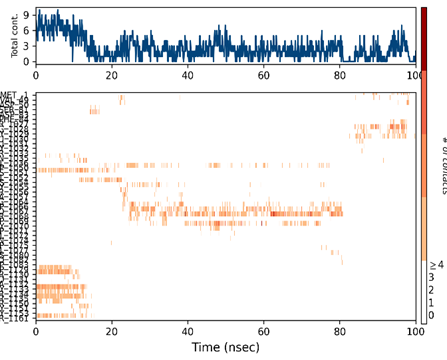 |
| --- | --- |
| (a) | (b) |
| 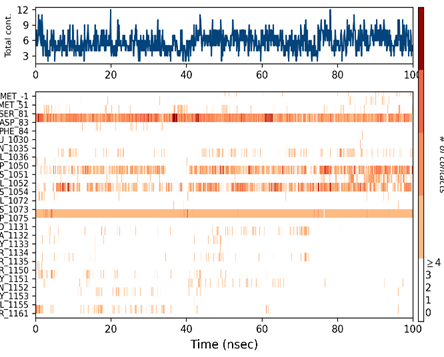 | 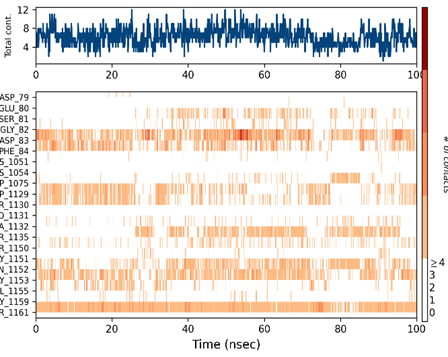 |
| (c) | (d) |
|  |  |

**Fig A5.** Comparison of Protein-Compound Contacts Timeline for Different Compounds. (a). Reference Compound. (b). Compound 68734190. (c) Compound 44418637. (d). Compound 163078083.

| 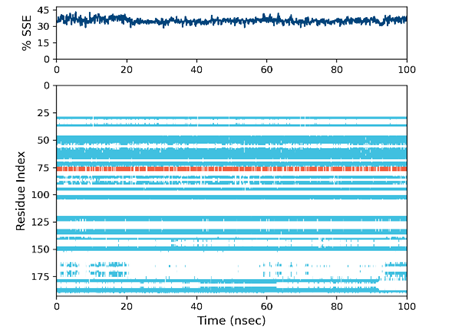 | 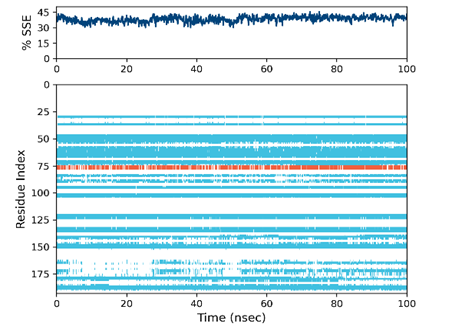 |
| --- | --- |
| (a) | (b) |
| 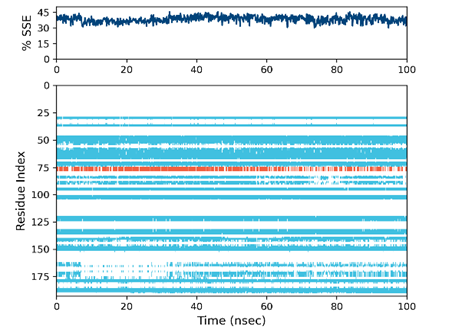 | 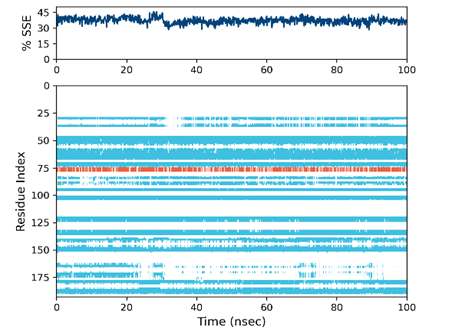 |
| (c) | (d) |

**Fig A6.** Comparison of Secondary Structure Timeline for Different Compounds (a). Reference Compound. (b). Compound 68734190. (c). Compound 44418637. (d). Compound 163078083.

**Table A1.** Details of the interactions of the top docked ligands with the binding site.

| Compound CID | H-bond | Distance (Å) | Electrostatic | Hydrophobic |
| --- | --- | --- | --- | --- |
| 166479806 | Asp83, Val1036, His1051, Ser1135, Tyr1161, Gly1151, Gly1133, Pro1131, Tyr1130, Thr1034 | 1.7-2.9 | / | His1051 Ala1132 Tyr1161 Tyr1130 |
| 166625687 | Asp83, Asn1152, Asp1075, Gly1151, Ser81 Val1072, Phe84, Gly82, His1051 | 1.6-2.9 | Asp1075, Asp83 | Tyr1161 Ala1132 Val1072 Val1154 |
| 68734254 | Gly1133, Ser1135, Val1036, His1051 Tyr1161, Tyr1130, Ala1132, Gly1151 | 1.8-3.1 | / | His1051 Tyr1161  Tyr1130 |
| 44418637 | Asp83, Asp1075, His1051, Lys1054, Gly1151, Tyr1161, Ser81, Val1072, Trp1050 | 1.7-2.9 | Lys1054, Asp83 Asp1075 | Ala1132 Tyr1161 Trp1050 Val1072 Lys1054 |
| 163078083 | Phe84, Asp83, Asn1152 Gly1153 | 1.8-2.8 | / | Tyr1161 |
| 42605183 | Ser1135, His1051 Asn1152, Phe84, Tyr1130 | 1.7-3.0 | / | Tyr1161 |
| 68734190 | Gly1133, Val1036, Tyr1130, Ala1132, Ser1135, Tyr1150 | 1.9-2.6 | / | His1051 |
| 58178603 | Gly1133, Gly1153, Asp83, Ala1132, Asn1152, Ser1135, His1051, Tyr1161 Thr1034, Phe84 | 1.7-2.9 | / | His1051, Tyr1161, Ala1132, Val1036 |

**Table A2.** ADME analysis of top docked ligands.

| Parameters | 166479806 | 166625687 | 68734254 | 44418637 | 163078083 | 42605183 | 68734190 | 58178603 | 137348520 |
| --- | --- | --- | --- | --- | --- | --- | --- | --- | --- |
| Mol-weight (g/mol) | 416.42 | 483.53 | 400.38 | 365.43 | 360.31 | 344.36 | 386.35 | 428.43 | 510.39 |
| H-acceptors | 8 | 8 | 8 | 5 | 8 | 6 | 8 | 8 | 7 |
| H-donors | 5 | 3 | 4 | 5 | 5 | 5 | 4 | 5 | 6 |
| Rotatable bonds | 4 | 10 | 3 | 12 | 3 | 4 | 4 | 4 | 14 |
| TPSA (Å 2) | 136.68 | 104.73 | 129.59 | 136.21 | 140.59 | 114.29 | 129.59 | 140.59 | 187.31 |
| Log S (ESOL) | -4.05 | -3.57 | -3.75 | -1.08 | -2.72 | -3.73 | -3.68 | -3.99 | -2.19 |
| Solubility (mg/ml) | 3.68e-02 | 1.31e-01 | 7.14e-02 | 5.76e+00 | 6.84e-01 | 6.41e-02 | 8.08e-02 | 4.41e-02 | 3.26e+00 |
| Class | Moderately soluble | Soluble | Soluble | Very  soluble | Soluble | Soluble | Soluble | Soluble | Soluble |
| GI absorption | High | High | High | High | High | High | High | High | Low |
| BBB permeant | No | No | No | No | No | No | No | No | No |
| P-gp substrate | Yes | Yes | Yes | Yes | No | Yes | No | Yes | No |
| CYP1A2  inhibitor | No | No | No | No | No | No | No | No | No |
| CYP2C19  inhibitor | No | No | No | No | No | No | No | No | No |
| CYP2C9  inhibitor | No | No | No | No | No | No | No | No | No |
| CYP2D6  inhibitor | No | No | No | No | No | Yes | No | No | No |
| CYP3A4  inhibitor | No | Yes | No | No | No | No | No | No | No |
| Lipinski | Yes | Yes | Yes | Yes | Yes | Yes | Yes | Yes | No |
| Veber | Yes | Yes | Yes | No | No | Yes | Yes | No | No |
| Bioavailability | 0.55 | 0.55 | 0.55 | 0.55 | 0.55 | 0.55 | 0.55 | 0.55 | 0.17 |
| Consensus  Log Po/w | 1.97 | 1.22 | 1.52 | 0.78 | 0.75 | 2.23 | 1.33 | 1.59 | -0.30 |
| Synthetic accessibility | 4.68 | 4.46 | 5.18 | 3.52 | 3.93 | 3.78 | 4.93 | 5.15 | 4.85 |

**Table A3.** Toxicity properties of selected compounds (A: Active / I: Inactive).

| Compound CID | Hepatotoxicity | Carcinogenicity | Immunotoxicity | Mutagenicity | Cytotoxicity | LD50 (mg/kg) |
| --- | --- | --- | --- | --- | --- | --- |
| 166479806 | I | I | I | I | I | 1000 |
| 68734254 | I | A | A | I | I | 5000 |
| 44418637 | I | I | I | I | I | 3000 |
| 163078083 | I | I | I | I | I | 562 |
| 68734190 | I | I | I | I | I | 5000 |
| 58178603 | I | I | I | A | A | 5000 |
| 137348520 | I | I | I | I | I | 550 |

**Table A4.** Bioactivity prediction of safety compounds.

| Compound CID | Protein target | Confidence |
| --- | --- | --- |
| 166479806 | Replicase polyprotein 1ab  Human immunodeficiency virus type 2 integrase | 0.8047  0.6363 |
| 44418637 | Dengue virus type 2 NS3 protein  Human immunodeficiency virus type 2 pol protein | 0.1061  0.0572 |
| 163078083 | Replicase polyprotein 1ab  Human immunodeficiency virus type 2 integrase | 0.6985  0.5837 |
| 68734190 | Replicase polyprotein 1ab  Human immunodeficiency virus type 2 integrase | 0.9781  0.3649 |
