# Supplementary material for: Identification and Evaluation of Natural Compounds as Potential Inhibitors of NS2B-NS3 Zika Virus Protease: A Computational Approach
Source: Mol Biotechnol. 2024 Dec 28;67(12):4632–50. doi: 10.1007/s12033-024-01357-6 (PMC12634716; doi:10.1007/s12033-024-01357-6)
Supplement: Supplementary file 2 — Supplementary file2 (DOCX 166 KB) [file 12033_2024_1357_MOESM2_ESM.docx]

**Appendix B**

**Identification and Evaluation of Naturel Compounds as Potential inhibitors of NS2B-NS3 Zika Virus protease: A Computational Approach**

**Nada Anede ^1^, Mebarka Ouassaf ^1,^ *, Kannan RR Rengasamy ^2,3^, Shafi Ullah Khan ^4,5^, and Bader Y. Alhatlani ^6,^ ***

^1^ Group of Computational and Medicinal Chemistry, LMCE Laboratory, University of Biskra, Biskra, Algeria; [nada.anede@univ-biskra.dz](mailto:nada.anede@univ-biskra.dz); [nouassaf@univ-biskra.dz](mailto:nouassaf@univ-biskra.dz)

^2^ Laboratory of Natural Products and Medicinal Chemistry (LNPMC), Saveetha Medical College and Hospital, Saveetha Institute of Medical and Technical Sciences (SIMATS), Thandalam, Chennai, 602105 India. [Ragupathi.Rengasamy@nwu.ac.za](mailto:Ragupathi.Rengasamy@nwu.ac.za)

^3^ Centre of Excellence for Pharmaceutical Sciences, North-West University, Potchefstroom, 2520, South Africa

^4^ UNICAEN, Inserm U1086 ANTICIPE (Interdisciplinary Research Unit for Cancer Prevention and Treatment), Normandie Univ, Caen, France; [shafiullahpharmd@gmail.com](mailto:shafiullahpharmd@gmail.com)

^5^ Cancer Centre François Baclesse, UNICANCER, Caen, France ; [shafiullahpharmd@gmail.com](mailto:shafiullahpharmd@gmail.com)

^6^ Unit of Scientific Research, Applied College, Qassim University, Buraydah 52571, Saudi Arabia; [balhatlani@qu.edu.sa](mailto:balhatlani@qu.edu.sa)

***** Correspondence: [balhatlani@qu.edu.sa](mailto:balhatlani@qu.edu.sa) (B.Y.A.); [nouassaf@univ-biskra.dz](mailto:nouassaf@univ-biskra.dz) (M.O.); [Ragupathi.Rengasamy@nwu.ac.za](mailto:Ragupathi.Rengasamy@nwu.ac.za) (KRRR).

Table B1. Different parameter scores of Enrichment Report

| ROC | BEDROC (α=160.9) | BEDROC (α=20) | BEDROC (α=8.0) | RIE | AUC |
| --- | --- | --- | --- | --- | --- |
| 0.91 | 0.137 | 0.512 | 0.667 | 8.10 | 0.89 |

Table B2: Count and percentage of actives in top N% of decoy results.

| %Decoys | 1% | 2% | 5% | 10% | 20% |
| --- | --- | --- | --- | --- | --- |
| #Actives | 1 | 7 | 16 | 18 | 21 |
| %Actives | 4.0 | 20.0 | 60.0 | 72.0 | 84.0 |

Table B3: Count and percentage of actives in top N% of results.

| %Results | 1% | 2% | 5% | 10% | 20% |
| --- | --- | --- | --- | --- | --- |
| #Actives | 1 | 5 | 15 | 18 | 21 |
| %Actives | 4.0 | 20.0 | 60.0 | 72.0 | 84.0 |

Table B4: Enrichment Factors with respect to N% actives recovered.

| %Actives | 40% | 50% | 60% | 70% | 80% | 90% | 100% |
| --- | --- | --- | --- | --- | --- | --- | --- |
| EF | 14 | 13 | 12 | 7.5 | 5.5 | 2.3 | 2.4 |
| EF* | 20 | 23 | 17 | 8.9 | 6.2 | 3.7 | 2.4 |
| EF' | 8.8 | 9.4 | 12 | 12 | 9.3 | 6.3 | 4.1 |
| FOD | 0.01 | 0.02 | 0.02 | 0.03 | 0.03 | 0.05 | 0.09 |

Total actives: 25

Total ligands (actives+decoys): 1025

Number of ranked actives: 25


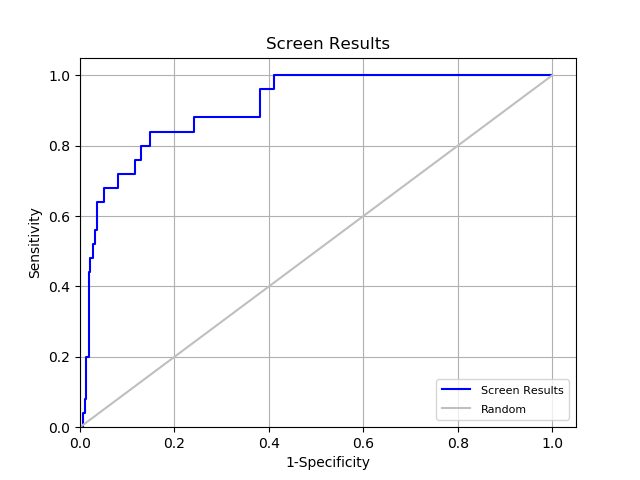


Fig B1: Plot map of the Receiver operating characteristic curve (ROC).

Table B5: NS2B-NS3 ZIKV protease Hits from screening

| Compound Names CID | Molecular Formula | Structures | PubChem IUPAC name | SMILES |
| --- | --- | --- | --- | --- |
| 166479806 | C_22_H_24_O_8_ | 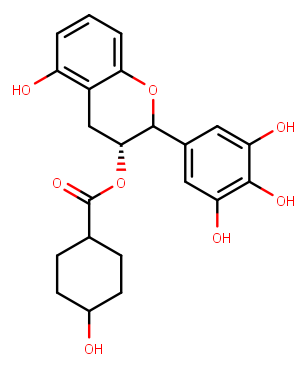 | [(3R)-5-hydroxy-2-(3,4,5-trihydroxyphenyl)-3,4-dihydro-2H-chromen-3-yl] 4-hydroxycyclohexane-1-carboxylate | C1CC(CCC1C(=O)OC2CC3=C(C=CC=C3OC2C4=CC(=C(C(=C4)O)O)O)O)O |
| 166625687 | C_26_H_31_N_2_O_7_^+^ | 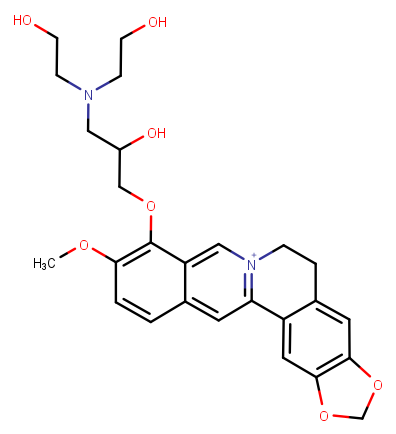 | 1-[bis(2-hydroxyethyl)amino]-3-[(17-methoxy-5,7-dioxa-13-azoniapentacyclo[11.8.0.02,10.04,8.015,20]henicosa-1(13),2,4(8),9,14,16,18,20-octaen-16-yl)oxy]propan-2-ol | COC1=C(C2=C[N+]3=C(C=C2C=C1)C4=CC5=C(C=C4CC3)OCO5)OCC(CN(CCO)CCO)O |
| 68734254 | C_21_H_20_O_8_ | 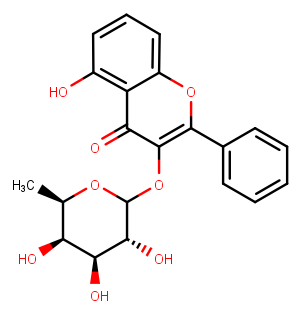 | 5-hydroxy-2-phenyl-3-[(3R,4S,5R,6R)-3,4,5-trihydroxy-6-methyloxan-2-yl]oxychromen-4-one | CC1C(C(C(C(O1)OC2=C(OC3=CC=CC(=C3C2=O)O)C4=CC=CC=C4)O)O)O |
| 44418637 | C_17_H_27_N_5_O_4_ | 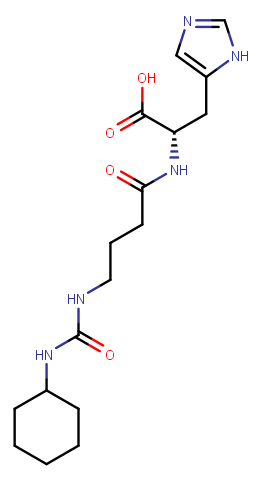 | (2S)-2-[4-(cyclohexylcarbamoylamino)butanoylamino]-3-(1H-imidazol-5-yl)propanoic acid | C1CCC(CC1)NC(=O)NCCCC(=O)NC(CC2=CN=CN2)C(=O)O |
| 163078083 | C_18_H_16_O_8_ | 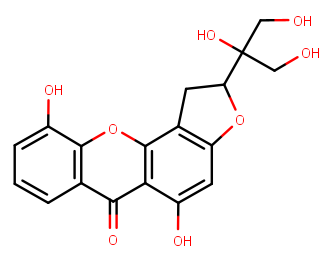 | 5,10-dihydroxy-2-(1,2,3-trihydroxypropan-2-yl)-1,2-dihydrofuro[2,3-c]xanthen-6-one | C1C(OC2=C1C3=C(C(=C2)O)C(=O)C4=C(O3)C(=CC=C4)O)C(CO)(CO)O |
| 42605183 | C_19_H_20_O_6_ | 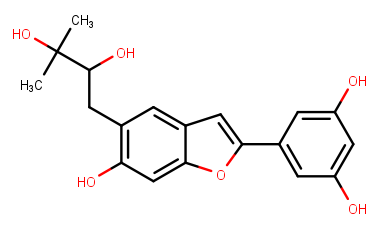 | 5-[5-(2,3-dihydroxy-3-methylbutyl)-6-hydroxy-1-benzofuran-2-yl]benzene-1,3-diol | CC(C)(C(CC1=C(C=C2C(=C1)C=C(O2)C3=CC(=CC(=C3)O)O)O)O)O |
| 68734190 | C_20_H_18_O_8_ | 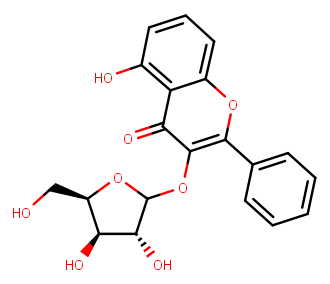 | 3-[(3R,4R,5R)-3,4-dihydroxy-5-(hydroxymethyl)oxolan-2-yl]oxy-5-hydroxy-2-phenylchromen-4-one | C1=CC=C(C=C1)C2=C(C(=O)C3=C(C=CC=C3O2)O)OC4C(C(C(O4)CO)O)O |
| 58178603 | C_23_H_24_O_8_ | 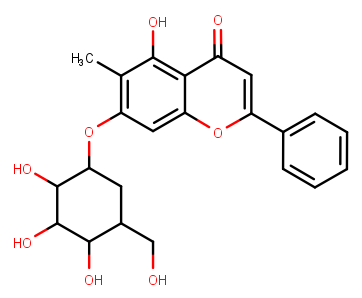 | 5-hydroxy-6-methyl-2-phenyl-7-[2,3,4-trihydroxy-5-(hydroxymethyl)cyclohexyl]oxychromen-4-one | CC1=C(C=C2C(=C1O)C(=O)C=C(O2)C3=CC=CC=C3)OC4CC(C(C(C4O)O)O)CO |

Table B6: molecular docking of hits compounds

| Groups | Compound CID | Docking score | Glide emodel | XP Gscore |
| --- | --- | --- | --- | --- |
| Epigallocatechin gallate | 166479806 | -8.454 | -60.980 | -8.459 |
|  | 102020683 | -8.282 | -56.751 | -8.282 |
|  | 10047283 | -8.169 | -64.354 | -8.169 |
| Berberine | 166625687 | -6.992 | -74.687 | -7.110 |
|  | 166625540 | -6.981 | -67.662 | -7.040 |
|  | 76311798 | -6.687 | -56.648 | -6.687 |
| Rutin | 68734254 | -7.750 | -61.548 | -7.752 |
|  | 22297706 | -7.716 | -47.690 | -7.736 |
|  | 163183160 | -7.670 | -70.803 | -7.679 |
| Carnosine | 44418637 | -7.336 | -56.995 | -7.721 |
|  | 73353617 | -6.894 | -48.419 | -7.466 |
|  | 14484048 | -6.858 | -50.253 | -7.370 |
| Myricetin | 146468892 | -8.117 | -49.169 | -8.149 |
|  | 58076365 | -8.426 | -61.530 | -8.426 |
|  | 163078083 | -8.226 | -48.204 | -8.226 |
| Hydroxypanduratin | 42605183 | -8.325 | -58.037 | -8.330 |
|  | 162842717 | -7.484 | -63.416 | -7.494 |
|  | 92030426 | -7.309 | -51.182 | -7.309 |
| Schaftoside | 4656410 | -8.609 | -52.935 | -8.609 |
|  | 68734190 | -8.305 | -63.170 | -8.307 |
|  | 140915838 | -8.197 | -53.883 | -8.236 |
| Silychristin | 58178603 | -9.165 | -68.635 | -9.166 |
|  | 163023241 | -8.649 | -61.696 | -8.650 |
|  | 76311690 | -8.471 | -65.916 | -8.481 |
